# CID12261165, a flavonoid compound as antibacterial agents against quinolone-resistant *Staphylococcus aureus*

**DOI:** 10.1038/s41598-023-28859-8

**Published:** 2023-01-31

**Authors:** Yuh Morimoto, Yoshifumi Aiba, Kazuhiko Miyanaga, Tomomi Hishinuma, Longzhu Cui, Tadashi Baba, Keiichi Hiramatsu

**Affiliations:** 1 Department of Radiological Technology, Faculty of Health Science, Juntendo University, 2-1-1 Hongo, Bunkyo-ku, Tokyo, 113-8421 Japan; 2 Division of Bacteriology, Jichi Medical School, 3311-1 Yakushiji, Shimotsuke-shi, Tochigi, 329-0498 Japan; 3 Department of Microbiology, School of Medicine, Juntendo University, Tokyo, Japan; 4 Graduate School of Nursing, Seisen Jogakuin College, 2277 Kurita, Nagano, 380-0921 Japan; 5 Juntendo University Center of Excellence for Infection Control Science, 2-1-1 Hongo, Bunkyo-ku, Tokyo, 113-8421 Japan

**Keywords:** Antibiotics, Antimicrobial resistance

## Abstract

Flavonoids are plant-produced secondary metabolites that are found ubiquitously. We have previously reported that apigenin, a class of flavonoid, has unique antimicrobial activity against *Staphylococcus aureus* (*S. aureus*), one of the major human pathogens. Apigenin inhibited fluoroquinolone-resistant *S. aureus* with DNA gyrase harboring the quinolone-resistant S84L mutation but did not inhibit wild-type DNA gyrase. In this study, we describe five flavonoids, quercetin, luteolin, kaempferol, baicalein, and commercially available CID12261165, that show similar antimicrobial activity against fluoroquinolone-resistant *S. aureus*. Among them, CID12261165 was the most effective with MIC values of ≤ 4 mg/L against quinolone-resistant *S. aureus* strains. In vitro DNA cleavage and supercoiling assays demonstrated inhibitory activity of CID12261165 against mutated DNA gyrase, whereas activity against wild-type DNA gyrase was not observed. CID12261165 also inhibited quinolone-resistant *Enterococci* with an MIC value of 8 mg/L. While fluoroquinolone-resistant amino acid replacements can improve the fitness of bacterial cells, it is unknown why quinolone-susceptible *S. aureus* strains were predominant before the introduction of fluoroquinolone. The present study discusses the current discrepancies in the interpretation of antimicrobial activities of flavonoids, as well as the possible reasons for the preservation of wild-type DNA gyrase wherein the environmental flavonoids cannot be ignored.

## Introduction

Fluoroquinolones are broad-spectrum synthetic antibacterial agents which were introduced in the early 1980’s^1^. Today, fluoroquinolones are used world-wide, and quinolone-resistant (QR) strains, including methicillin-resistant *S. aureus* (MRSA), have emerged globally^2^. *S. aureus* is one of the ESKAPE pathogens [*Enterococcus faecium* (*E. faecium*), *Staphylococcus aureus* (*S. aureus*), *Klebsiella pneumoniae* (*K. pneumoniae*), *Acinetobacter baumannii* (*A. baumannii*), *Pseudomonas aeruginosa* (*P. aeruginosa*), and *Enterobacter* species], whose emergence of antimicrobial resistance, including fluoroquinolone resistance, is a global threat^3,4^. The most common QR mechanism is mutation(s) occurring in DNA gyrase and topoisomerase IV^2,3^. Amino acid substitutions of *parC* Ser80 and *gyrA* Ser84, or corresponding sites, alter the target protein structure reducing fluoroquinolone binding affinity^2,5^. Unlike acquisition of β-lactam or vancomycin resistance^6^, QR mutations do not compromise microbial growth. Multiple mutations in DNA gyrase show fitness of similar or even greater level than that with quinolone susceptible (QS) gyrase^7^. Recently, fluoroquinolones have been considered involved in selection for major international clones/STs of various multi-drug resistant pathogens commonly found with multiple QR mutations^5^.

Flavonoids are plant-produced secondary metabolites that are found ubiquitously^8^. We previously reported that apigenin, a class of flavonoids, showed antimicrobial activity against QR *S. aureus*^9^. Apigenin inhibits DNA gyrase harboring S84L mutation in *gyrA* but does not inhibit wild-type DNA gyrase^9^. Similar to our earlier study of nybomycin^10^, apigenin resistant mutant selection generated L84S back mutations, providing re-acquisition of fluoroquinolone susceptibility^9^.

In this study, we evaluated the minimum inhibitory concentration (MIC) of five flavonoids, quercetin, luteolin, kaempferol, baicalein, and commercially available CID12261165, against ESKAPE pathogens. Subsequently, we focused the mode of action, and selection of resistant mutants of CID12261165 against QR *S. aureus* and describe the potential impact of flavonoids on bacterial DNA gyrase. The present study discusses the environmental factors influencing human pathogens and their drug targets. We also outline the current discrepancies in the interpretation of the antimicrobial activities of flavonoids.

## Results

### Anti-quinolone resistance activity of the five flavonoids against QS and QR *S. aureus* strains

Antibacterial activities of the five flavonoids were evaluated against six *S. aureus* strains. Of them, two strains were QS methicillin-susceptible *S. aureus* (MSSA) strains (FDA 209P and NCTC 8325), one strain was QR MRSA strain USA300FPR, one strain was QR vancomycin-intermediate *S. aureus* (VISA) strain Mu50, and two strains were QR vancomycin-resistant *S. aureus* (VRSA) strain VRS1 and VRS5. While all tested flavonoids showed weak or no activity against QS *S. aureus* (32 to >128mg/L), they inhibited bacterial growth of QR *S. aureus* strains with MIC values of 2–16 mg/L, except baicalein against VRS1 (32 mg/L) (Table 1). CID12261165 showed the most significant antibiotic activity against QR *S. aureus* with an MIC value equivalent or greater to that of apigenin.

**Table 1:** Minimum inhibitory concentration of the tested flavonoids against *S. aureus* strains with various QRDR mutations.

		QRDR mutation(s)		MIC (mg/L)						
		*parC*	*gyrA*	CID12261165	Quercetin	Luteolin	Kaempferol	Baicalein	Apigenin	Levofloxacin
FDA 209P	QS-MSSA	–	–	128	64	32	32	32	128	**0.125**
NCTC 8325	QS-MSSA	–	–	128	32	32	> 128	64	> 128	**0.125**
USA300 FPR	QR-MRSA	S80Y	S84L	**4**	16	16	8	8	16	**4**
Mu50	QR-VISA	S80F	S84L	**4**	16	16	16	16	**4**	8
VRS1	QR-VRSA	S80F	S84L	**4**	16	8	8	32	8	16
VRS5	QR-VRSA	S80Y, E84G	S84L, E88K	**2**	8	8	8	8	8	32

*QS* quinolone susceptible, *QR* quinolone resistant, *MSSA* methicillin-susceptible *S. aureus*, *MRSA* methicillin-resistant *S. aureus*, *VISA* vancomycin-intermediate *S. aureus*, *VRSA* vancomycin-resistant *S. aureus*. MICs ≤ 4 mg/L are in bold.

To investigate the relationship between CID12261165 susceptibility and quinolone resistance mutations, MRSA strain MR5867 and its stepwise quinolone mutated strains were chosen. Firstly, the doubling time of MR5867 and its variants were measured. Doubling time was increased with acquisition of a single mutation in *parC* and *gyrA*, however it recovered after acquiring second mutations in *parC* and *gyrA* (Table 2, Supplemental Table 1). Subsequently, MIC values of CID12261165, apigenin, and levofloxacin were measured. Similar to apigenin, CID12261165 showed no antimicrobial activity against MR5867 parent strain, however the MIC value dropped from 128 to 4 mg/L upon acquisition of S84L mutation in *gyrA* (Table 2).

**Table 2:** MIC values of *S. aureus* Quinolone stepwise mutant strain MR5867.

	Parent	1st step	2nd step	3rd step	4th step
*parC*	WT	E84K	E84K	S80F, E84K	S80F, E84K
*gyrA*	WT	WT	S84L	S84L	S84L, E88K
DT (min)	27.51	34.32	31.56	28.16	26.68
CID12261165	128	16	**4**	**4**	**4**
Apigenin	> 128	128	8	**4**	**4**
Levofloxacin	**0.125**	**0.5**	8	16	> 128

*DT* doubling time, *WT* wild type. MICs ≤ 4 mg/L are in bold.

### Anti-quinolone resistance activity of the five flavonoids against ESKAPE pathogens and *E. faecalis*

In addition to *S. aureus*, five other ESKAPE pathogens and *E. faecalis* were tested for their susceptibility against the flavonoids for both QS and QR strains. CID12261165 and luteolin showed antimicrobial activity against QR *Enterococcus* with MIC values of 8 mg/L and 16 mg/L, respectively. The MIC values of CID12261165 and luteolin against all other strains were ≥ 32 mg/L. The MIC values of quercetin, kaempferol, and baicalein against all tested strains were ≥ 32 mg/L, whether or not the strain was QS or QR (Table 3).

**Table 3:** Minimum inhibitory concentration of the flavonoids against QS and QR ESKAPE pathogens besides *S. aureus* and *E. faecalis* strains.

			QRDR mutation(s)		MIC (mg/L)							
			*parC*	*gyrA*	CID12261165		Quercetin	Luteolin	Kaempferol	Baicalein	Apigenin	Levofloxacin
*E. faecalis*	QS	NCTC12201	–	–	128		128	128	128	128	128	**1**
	QR	36–15722	S82I	S84I	8		128	16	32	64	16	64
*E. faecium*	QS	NCTC12202	–	–	128		128	128	128	128	128	**1**
	QR	Rhône-Poulenc 2	S82I	S84I, E88A	8		64	16	32	128	64	128
*P. aeruginosa*	QS	PAO1	–	–	64		64	64	64	64	64	**0.125**
	QR	JICC 50004	S87L	S80I	32		32	32	32	32	32	> 64
*E. cloacae*	QS	1498	–	–	> 64		> 64	> 64	> 64	> 64	> 64	** ≤ 0.06**
	QR	1505	S80I	S83I	> 64		> 64	> 64	> 64	64	> 64	> 64
*K. pneumoniae*	QS	ATCC 9997	–	–	32		64	64	64	64	64	** ≤ 0.06**
	QR	50024	S80I	S83F, D87A	> 64		> 64	> 64	> 64	64	> 64	8
*A. baumannii*	QS	1404	–	–	64		> 64	> 64	> 64	64	> 64	** ≤ 0.06**
	QR	1429	S80L	S83L	64		64	64	64	64	64	8

*QS* quinolone susceptible, *QR* quinolone resistant.

MICs ≤ 4 mg/L are in bold.

### Inhibition of mutated DNA gyrase by CID12261165

**Figure 1:**
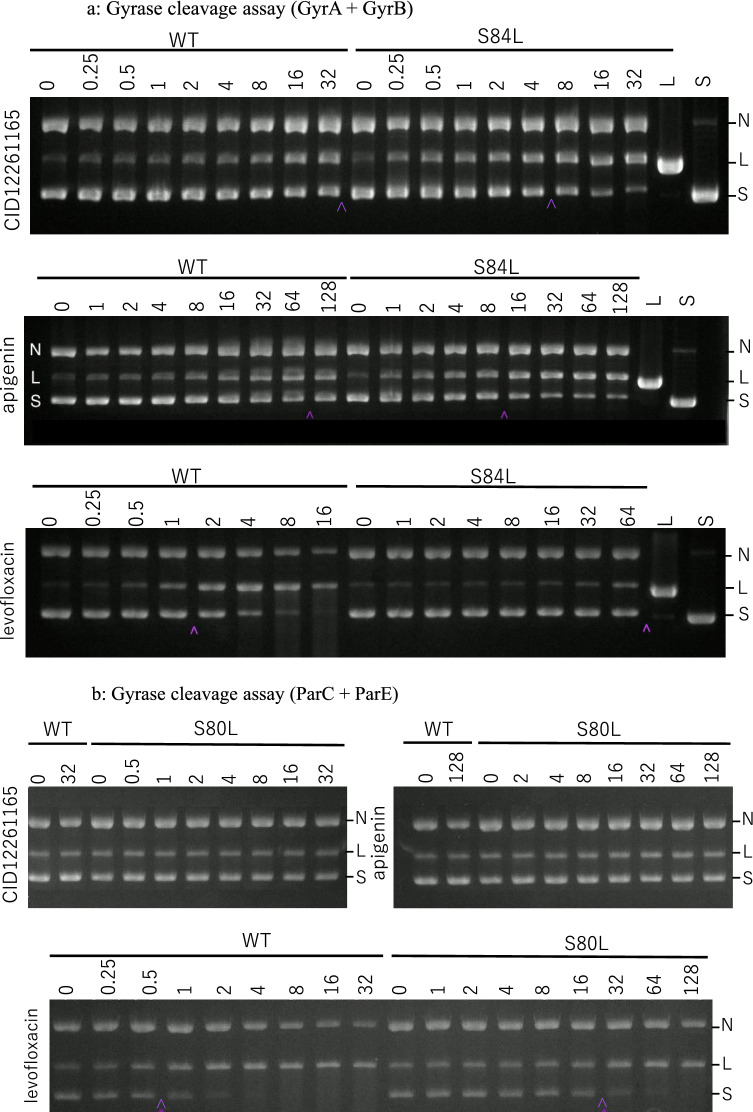

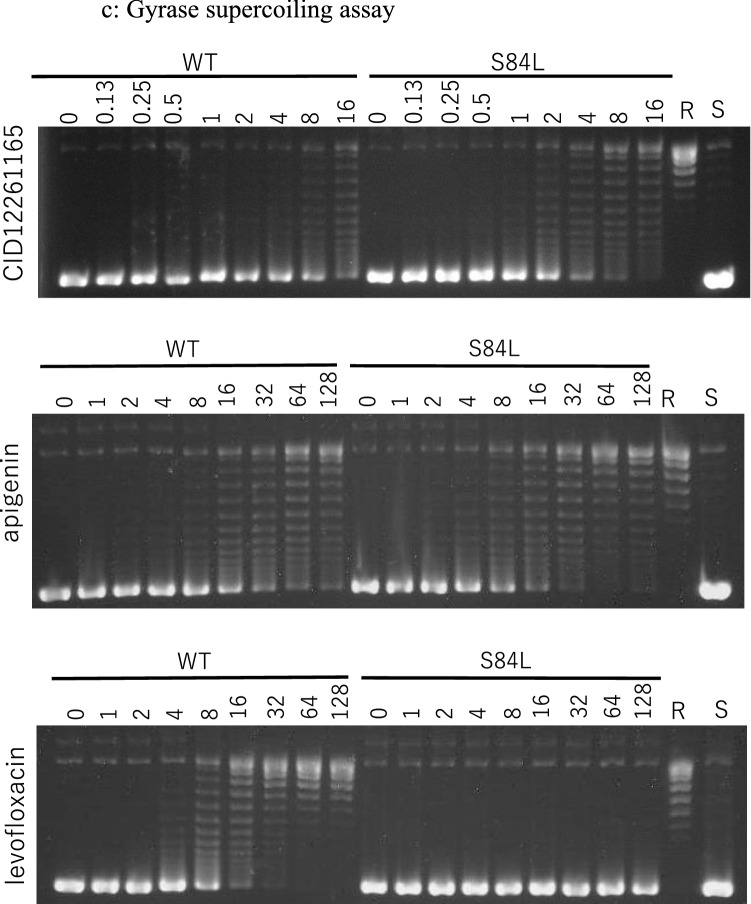
**(a)** In vitro DNA cleavage assay showing CID12261165 and apigenin specifically inhibited mutated DNA gyrase, whereas the mutant was resistant to levofloxacin. In the figure, ‘S’ indicates supercoiled substrate plasmid, ‘L’ is the linearized form of the plasmid, and ‘N’ indicates the nicked form of the plasmid. Decreased population of ‘S’ and corresponding accumulation of ‘L’ and ‘N’ forms indicate failure of DNA re-ligation activity by effective drugs after cleavage of the substrate DNA by DNA gyrase. (**b)** Whereas levofloxacin inhibited enzymic activity of topoisomerase IV, CID12261165 and apigenin neither inhibited mutated or wild-type topoisomerase IV. (**c)** In vitro DNA supercoiling assay showing enzymatic activity of CID12261165 and apigenin mutated form of DNA gyrase, whereas the mutant was resistant to levofloxacin. In the figure, ‘R’ indicates relaxed plasmid (substrate), and ‘S’ is the supercoiled form of the plasmid (reaction product). WT: wild-type GyrA + GyrB; S84L: GyrA(S84L) + wild-type GyrB. *S. aureus* strain FDA209P was the source of wild-type recombinant GyrA and ParC, and strain Mu50 was the source of recombinant S84L GryA and S80F ParC with one mutation, respectively. Decreased population of ‘S’ and corresponding accumulation of ‘R’ forms indicate failure of DNA supercoiling activity by effective drugs. Carets (hat symbol) in the (**a,b**) show IC50 values. Each image is cropped from gels by different experiments, and the original images are provided as supplemental materials.

To further investigate the antibiotic activity of CID12261165, the ability of the compound to inhibit DNA gyrase was determined using in vitro DNA cleavage assay. Similar to apigenin, CID12261165 inhibited S84L GyrA with an IC_50_ value of 4 mg/L, which was eightfold greater than that of wild-type GyrA (> 32 mg/L) (Fig. 1a). In contrast, CID12261165 and apigenin inhibited neither mutated nor wild-type topoisomerase IV, whereas levofloxacin inhibited wild-type topoisomerase IV with an IC_50_ value of 0.5 mg/L (Fig. 1b). DNA supercoiling assay demonstrated that CID12261165 and apigenin were likely to bind with a higher affinity to S84L GyrA compared to wild-type GyrA (Fig. 1c). In contrast, using levofloxacin as a compound of interest, failure of DNA supercoiling activity was decreased upon acquisition of S84L mutation in GyrA. Both DNA gyrase cleavage assay and supercoiling assay indicated CID12261165 was effective against S84L GyrA but not wild-type DNA gyrase.

### Development of resistance to CID12261165

**Figure 2:**
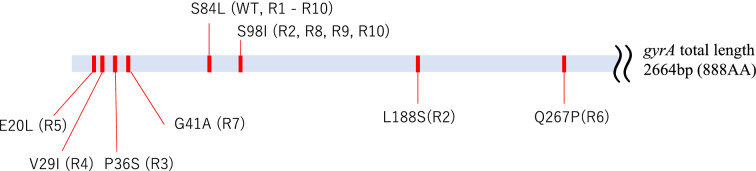
*S. aureus* strain Mu50 (WT) and CID12261165 mutant derivatives (R1 to R10) with various mutations in *gyrA.*

To explore whether CID12261165 susceptible *S. aureus* strain could develop resistance, mutant strains were selected from the CID12261165-containing agar plate. Ten independently cultured Mu50 strains were plated on 32 mg/L of CID12261165-containing agar plates with a total CFU between 1.90 × 10^7^ and 1.78 × 10^8^. During two-overnight incubation, three to 15 colonies grew on all 10 mutant-selection plates at an appearance rate of 5.79 × 10^–7^ to 2.80 × 10^–8^. One colony from each plate was isolated for further investigation. All resistant strains developed MIC values of CID12261165 > 64 mg/L. DNA sequencing confirmed the ten resistant strains retained S84L mutation and acquired an additional mutation in *gyrA*. S98I was the most common mutation (resistant strains 2, 8, 9, and 10), with mutations of E20L, V29I, P36S, G41A, L188S, and Q267P also observed in one strain each. Unlike apigenin, CID12261165 did not generate L84S reverse mutation to recover fluoroquinolone susceptibility (Fig. 2, Supplemental Table 2).

### In silico drug feature analysis of the flavonoids

The results of in silico drug profiling analyses are summarized in Table 4, and detailed reports are provided as supplemental material. All flavonoids tested were predicted to be cytochrome (CYP) P450 inhibitors with CYP1A2 ≥ 0.94. In addition to CYP1A2, CID12261165, luteolin, baicalein, and apigenin were predicted to inhibit CYP2C19, quercetin, luteolin, and apigenin were predicted to inhibit CYP3A4, and kaempferol and apigenin were predicted to inhibit CYP2C9. All tested flavonoids were also predicted to bind human plasma protein with the plasma protein binding (PPB) of > 90%. The Ames positive probability was high (> 0.85) in CID12261165, quercetin, kaempferol, and baicalein, while all tested flavonoids were predicted to be non-hERG-inhibitors.

**Table 4:** In-silico drug profiling analysis of the flavonoids.

	CID12261165	Quercetin	Luteolin	Kaempferol	Baicalein	Apigenin	Levofloxacin
Cytochrome P450 inhibitors (IC50 < 50 μM)							
CYP3A4	0.71	**0.83**	**0.92**	0.36	0.61	**0.93**	0.01
CYP2D6	0.15	0.23	0.15	0.28	0.05	0.13	0.06
CYP2C9	0.77	0.68	0.71	**0.93**	0.78	**0.90**	0.03
CYP2C19	**0.92**	0.37	**0.89**	0.66	**0.84**	**0.94**	0.02
CYP1A2	**0.95**	**0.95**	**0.95**	**0.94**	**0.94**	**0.94**	0.10
Plasma protein binding (PPB)							
Percentage bound to human plasma proteins	**91.03**	**93.38**	**93.51**	**93.54**	**90.94**	**94.02**	32.89
Toxicity prediction							
Ames positive probability	**0.82**	**0.85**	0.48	**0.92**	**0.94**	0.47	0.46
hERG inhibitor probability (Ki < 10 μM)	0.04	0.00	0.00	0.00	0.00	0.00	0.06

Bold in Cytochrome P450, protein binding, and Ames are inhibitor, > 90%, and mutagenic, respectively.

## Discussion

Flavonoids are estimated to have been present on Earth for around 470 million years, since the emergence of primitive land plants^8^. Biosynthesis of flavonoids is associated with environmental stresses, e.g., UV radiation, pathogens, and mineral nutrient imbalances^8,11^. Besides apigenin, we previously reported isopratol and myricetin-3,7,3′,4′-tetramethyl ether showed antibiotic activity against QR *S. aureus*^9^. Eight flavonoids that have been found so far to show activity against *S. aureus* showed stronger activity against QR strains than QS strains. While we have tested over 100 commercially available flavonoids, we have not found a flavonoid with antimicrobial activity against QS *S. aureus* strain FDA209P stronger than that against QR *S. aureus* strain Mu50 (Morimoto Y, unpublished data). Notwithstanding that QR mutations do not negate the fitness of bacterial cells, QS strains with S84 in *gyrA* were predominant before the introduction of fluoroquinolone^12^. In this study, doubling times were accelerated by acquisition of multiple mutations in *parC* and *gyrA*, while susceptibility against flavonoids increased. Our findings correspond with the assumption of Fuzi M*, *et al., that the current wild-type DNA gyrase sequence is the result of protection against environmental compounds^5,12^. One limitation is that the number of strains used in this study is relatively small. Further investigation of both QS and QR *S. aureus* strains should be conducted to confirm the antimicrobial efficacy of flavonoids, especially CID12261165.

Mutant selections of CID12261165 were performed using flavonoid-sensitive *S. aureus* strain Mu50, which was used in a previous study to select mutant strains against apigenin^9^. Despite our expectations, CID12261165 did not induce L84S reverse mutation but caused various amino-acid substitutions in GyrA. Note that, although apigenin did induce reverse mutation, it also induced several other mutations in *gyrA*, *gyrB*, *rsbU* and *murC*^9^. Unlike nybomycin which only generated reverse mutation^10^, mutations caused by flavonoids tolerate several alternative substitutions. Flavonoids may not only affect preserving S84 in bacterial *gyrA* but also maintaining genetic diversities of DNA gyrase, which may contribute to bacterial adaptation in different conditions, including multi-drug environments.

We have also tested flavonoids antimicrobial activities against five other ESKAPE pathogens and *E. faecalis*. Although CID12261165 showed mild antimicrobial activity against *E. faecium* and *E. faecalis*, none of the tested flavonoids were active against four other ESKAPE pathogens. Since those four species are Gram-negative strains, different mechanisms may be involved in the resistance in flavonoids, such as the efflux pump and the structure of the outer membrane.

Antibacterial activities of flavonoids against human pathogens have been studied for decades^13–21^. However, discrepancies between reports of flavonoid antibacterial activity have been indicated^13,22^, and inconsistent evaluation methods depending on the studies are considered to be resulting in such discrepancies^22^. In addition to methodological discrepancies, we have noticed that the interpretation of MIC data does not have agreement. According to Clinical and Laboratory Standards Institute guideline M100, the antibiotics commonly used for the treatment of *S. aureus* infection are determined susceptible ≤ 8 mg/L, and the majorities of them are ≤ 2 mg/L^23^. Therefore, we interpretated that MIC values ≥ 16 mg/L were ineffective. However, several studies propose the positive antimicrobial activity of flavonoids with a higher range^15,17,24–26^, which can be as high as g/L order^25,26^. Indeed, some of MIC evaluations were carried out in the body of the antibiofilm study, and interpretation of the MIC results may be open to some degree of flexibility depending on study aims. However, since pharmacological application of flavonoids is often discussed^27^, a general consensus on the interpretation of MIC values of flavonoids should be determined.

The results of our in silico analysis of cytochrome (CYP) P450 inhibition, PPB, and toxicity prediction were consistent with previously reported in vitro and in vivo studies. CYP enzymes are essential for the metabolism of many medications, and inhibition of CYP may lead to significant drug–drug interactions (DDI)^28,29^. CYP inhibitory activity of quercetin, kaempferol, and apigenin had been demonstrated by in-vitro studies of human CYP isoforms^30^, thus the in-silico results showing CID12261165 as a CYP inhibitor is considered to be reasonable. The quercetin conjugates were detected from human and rat plasma with a quercetin-rich diet^31,32^. Likewise, PPB prediction of all tested flavonoids were > 90%. Although the clinical significance of PPB is generally low, according to U.S. Department of Health and Human Services Food and Drug Administration, the possibility of displacement interactions from plasma protein binding sites should be assessed^29^. Predictions of the mutagenicity were positive in CID12261165, quercetin, kaempferol, and baicalein in our in silico analysis. Likewise, quercetin and kaempferol were reported to show mutagenicity on the in vitro Ames test elsewhere, whereas luteolin and apigenin were not^33,34^.

Many studies discuss opportunities for the pharmacological application of flavonoids^14,17,25,35–37^. However, our results suggest extra consideration is required for flavonoids to be applied as therapeutic agents, including in livestock environments which are currently abused by a tremendous amount of antibiotics^38^ and in water environments as antibiofilm substances, which require a larger amount of compounds compared to applications in﻿ vivo. Both CID12261165 in this study and apigenin in the previous study showed a tendency to induce multiple gyrase mutations^9^, which can influence the fitness cost of bacteria. Admission of anthropogenically purified flavonoids may cause the emergence of highly resistant bacteria with improved fitness. Unlike many other antibiotics that are produced by specific organisms, such as actinomycetes and fungi, flavonoids exist in the environment ubiquitously, thus, it is impossible to control the amount of environmental flavonoids once somehow it becomes necessary.

In this study, we demonstrated that the five flavonoids, quercetin, luteolin, kaempferol, baicalein, and commercially available CID12261165, inhibited fluoroquinolone-resistant *S. aureus* and Enterococci with DNA gyrase harboring the quinolone-resistant mutations to some extent but did not inhibit wild-type DNA gyrase. While QR mutation can improve the fitness of bacterial cells, environmental flavonoids may contribute to the evolutional preservation of wild-type DNA gyrase. Since there are possibilities for flavonoids to induce multiple gyrase mutations and to lead DDI, judicious strategies based on the One Health approach are warranted for the consideration of pharmacological applications.

## Materials and methods

### Flavonoid compounds

All flavonoid compounds used in this study are commercially available. PubChem CID12261165 (= Otava 1655041 = ZINC41195136) was from Otava chemicals MB (Vilniaus, Lithuania). Apigenin (A1514), quercetin (P0042), luteolin (T2682), kaempferol (K0018), and baicalein (T2721) were from Tokyo Chemical Industry Co., Ltd (Tokyo, Japan). Levofloxacin was from LKT Laboratories Inc (Minnesota, USA).

### Bacterial strains

The *S. aureus* strains used in this study, FDA209P (ATCC 6538P and NCTC 7447), Mu50 (ATCC700699), NCTC 8325, USA300FPR, VRS1, VRS5 were previously described^9,10^. MR5867 is a quinolone-susceptible methicillin-resistant *S. aureus* clinical isolate. The quinolone-resistant first- and second-step mutants were selected sequentially with norfloxacin and ofloxacin, and the third- and fourth-step mutants were obtained from the second-step mutants by sequential selection with 4× the MICs of tosufloxacin and sparfloxacin, respectively^39^. QS *E. faecalis* and *E. faecium* strains were NCTC12201 and NCTC12202 (QS), both from National Collection of Type Culture (Salisbury, UK). QR *E. faecalis* and *E. faecium* were clinical isolates of 36–15722 from Shionogi & Co., Ltd. and Rhône-Poulenc 2 from Rhône-Poulenc-Rorer, respectively^40^. *P. aeruginosa* PAO1 (NCRB106052) was obtained from National Institute of Technology, and strain JICC 50004 was clinically isolated from Juntendo University Hospital in 2006. *E. cloacae* strain 1498 and 1505 were clinically isolated in 2016 and provided by BML Inc. Japan. *K. pneumoniae* ATCC 9997 was obtained from American Type Culture Collection. *K. pneumoniae* 50024, *A. baumannii* 1404, *A. baumannii* 1429 were clinically isolated in 2009 and provided by Miroku Medical Laboratory (Japan).

### Antibiotic susceptibility test

Microdilution broth susceptibility tests were performed in accordance with Clinical and Laboratory Standards Institute guidelines^41^. Dimethyl sulfoxide (DMSO) was used to prepare 1024 mg/L flavonoid solutions, and sterile pure water was used to dissolve and prepare 1024 mg/L levofloxacin solution. The solutions were then diluted with cation-adjusted BD BBLTM Mueller–Hinton II Broth (Cation-Adjusted) (Becton Dickinson, MD, USA) for MIC measurement.

### Measurement of doubling time

The doubling time was calculated as described^42^. Overnight cultures of strain MR5867 were adjusted to approximately 10^8^ cfu/ml, then 10 μl of the diluted cultures were added to 10 ml fresh tryptic soy broth (Becton Dickinson, MD, USA) and grown at 37 °C with shaking at 25 rpm in an automatic photorecording incubator (TN-2612, Advantec, Tokyo, Japan). Optical density at 600 nm (OD600) was automatically monitored and recorded every 2 min. Doubling time measurements were performed in three independent experiments.

### Demonstration of gyrase inhibition by DNA cleavage assay and DNA supercoiling assay

*S. aureus* FDA209P was the source of wild-type recombinant DNA gyrase subunit A (GyrA) and DNA topoisomerase IV subunit C (ParC). Mu50 was the source of recombinant GryA and ParC with one mutation, S84L and S80F, respectively. The assay was based on an established system described by Fisher and Pan^43^. Briefly, supercoiled plasmid DNA pTWV228 (a pBR322 derivative) (TaKaRa Bio Inc., Kusatsu, Japan) was preincubated in the presence of compounds of interest, followed by addition of GyrB in combination with either wild-type or mutated GyrA proteins and then incubated at 25 °C for 1 h in DNA cleavage assay. Relaxed pBR322 plasmid DNA (Inspiralis Limited, Norwich, UK) was employed for supercoiling assay. The following analyses, including determination of IC_50_ values (50% inhibitory concentration) for the cleavage assays, were conducted as described^10^.

### CID12261165 mutant selection

Ten independently cultured Mu50 were plated on CID12261165 containing Mueller–Hinton agar plates (Becton Dickinson, MD, USA) with drug concentration of 32 mg/L. Plates were incubated at 37 °C for 48 h, then one colony from each was selected for further investigation. The inoculation size of ten mutant selection plates was estimated by counting the number of colonies inoculated on the drug-free Mueller–Hinton agar plate with serial dilution of the samples. The appearance rate of CID12261165 mutants was determined by dividing the number of colonies on a selection plate by the number of colonies on a drug-free plate.

### DNA sequencing of *gyrA* and *parC*

Two sets of forward and reverse primers, respectively were used for PCR amplification: 5′-GATTGAAGCGGACCAAACAT-3′ and 5′-TTTATTGGCGAAAACCTTGC-3′ for *S. aureus gyrA*; 5′-TTAGGTGATCGCTTTGGAAGATATAG-3′ and 5′-TACCATTGGTTCGAGTGTCG-3′ for *S. aureus parC*, 5′-CCGGTTAACATCGAGGAAGA-3′ and 5′-ATGTGTTCCATCAGCCCTTC-3′ for *E. cloacae gyrA*; and 5′-GAATTCACGGAAAACGCCTA-3′ and 5′-GCCGTTCTGGTAGATTTTGC-3′ for *E. cloacae parC*. The sequence primers for *P. aeruginosa*, *E. faecalis, E. faecium, K. pneumoniae* and *A. baumannii* were described elsewhere^44–48^. In addition, three forward primers were used to cover the whole *S. aureus gyrA* genes: 5′-GCCAATGGAGCATCAGGTAT-3′, 5′-GGTTTAGAGAGAGACAAAATTGAAGC-3′, and 5′-ACAATGATTGCTGTTAAAGACCTTG-3′. Sequencing of the amplified DNA was done using a BigDye Terminator v.3.1 Cycle Sequencing Kit (Applied Biosystems, Life Technologies, CA, USA) and ABI PRISM 3100 Genetic Analyzer (Applied Biosystems, Life Technologies, CA, USA).

### In silico drug profile analysis of the flavonoids

In silico drug profiling was performed using ACD/Percepta 14.3.0 (Advanced Chemistry Development Inc, Canada) using the applications’ default setting.

## Supplementary Information

Supplementary Information 1.

Supplementary Tables.

Supplementary Figure 1.

Supplementary Information 2.

## Data Availability

The DNA sequences for recombinant proteins of DNA gyrase and DNA topoisomerase IV subunits fused to maltose-binding protein used in this study are deposited in DDBJ/EMBL/GenBank databases with accession numbers as follows: wild-type GyrA (LC727689), GyrA with a mutation (LC727690), GyrB (LC727691), wild-type ParC (LC727692), ParC with a mutation (LC727693) and ParE (LC727694). The data that supports the findings of this study is available in the supplementary material of this article.
