# Supplementary material for: CID12261165, a flavonoid compound as antibacterial agents against quinolone-resistant Staphylococcus aureus
Source: Sci Rep. 2023 Jan 31;13:1725. doi: 10.1038/s41598-023-28859-8 (PMC9889749; doi:10.1038/s41598-023-28859-8)

Fig 1a middle →
(apigenin)

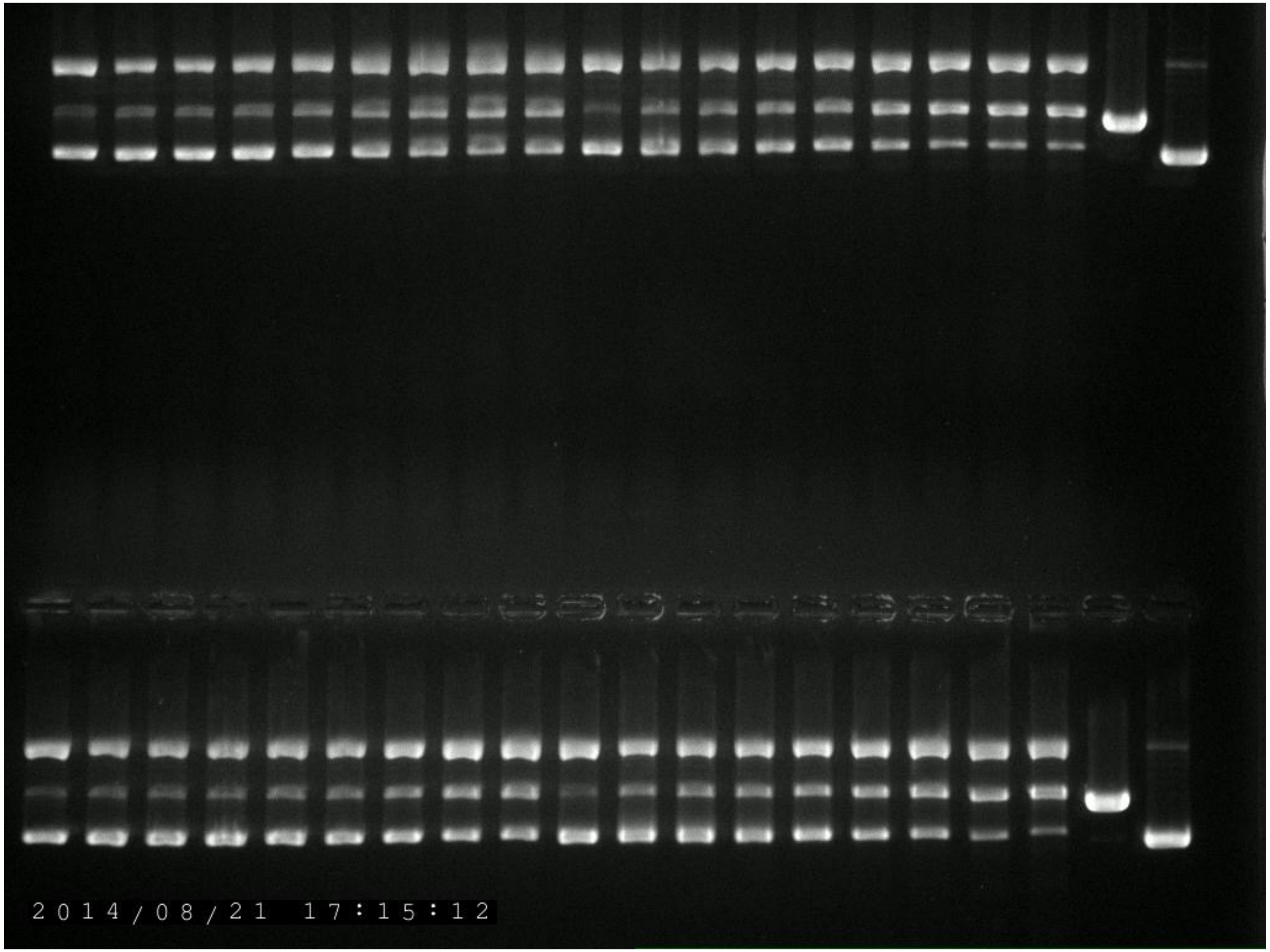

Fig 1a top →
(CID12261165)

Fig 1a bottom →
(levofloxacin)

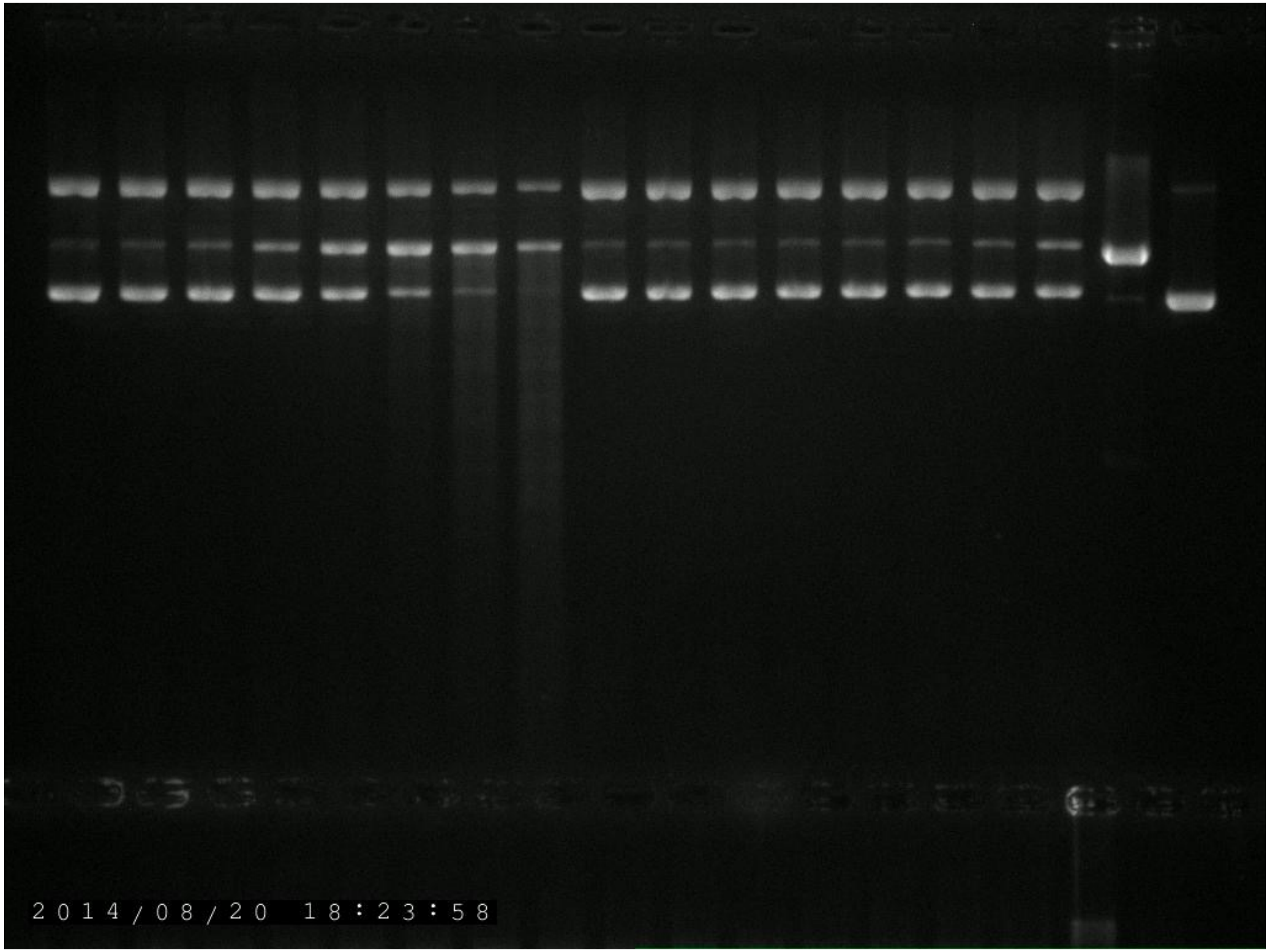

Fig 1b bottom → (levofloxacin)

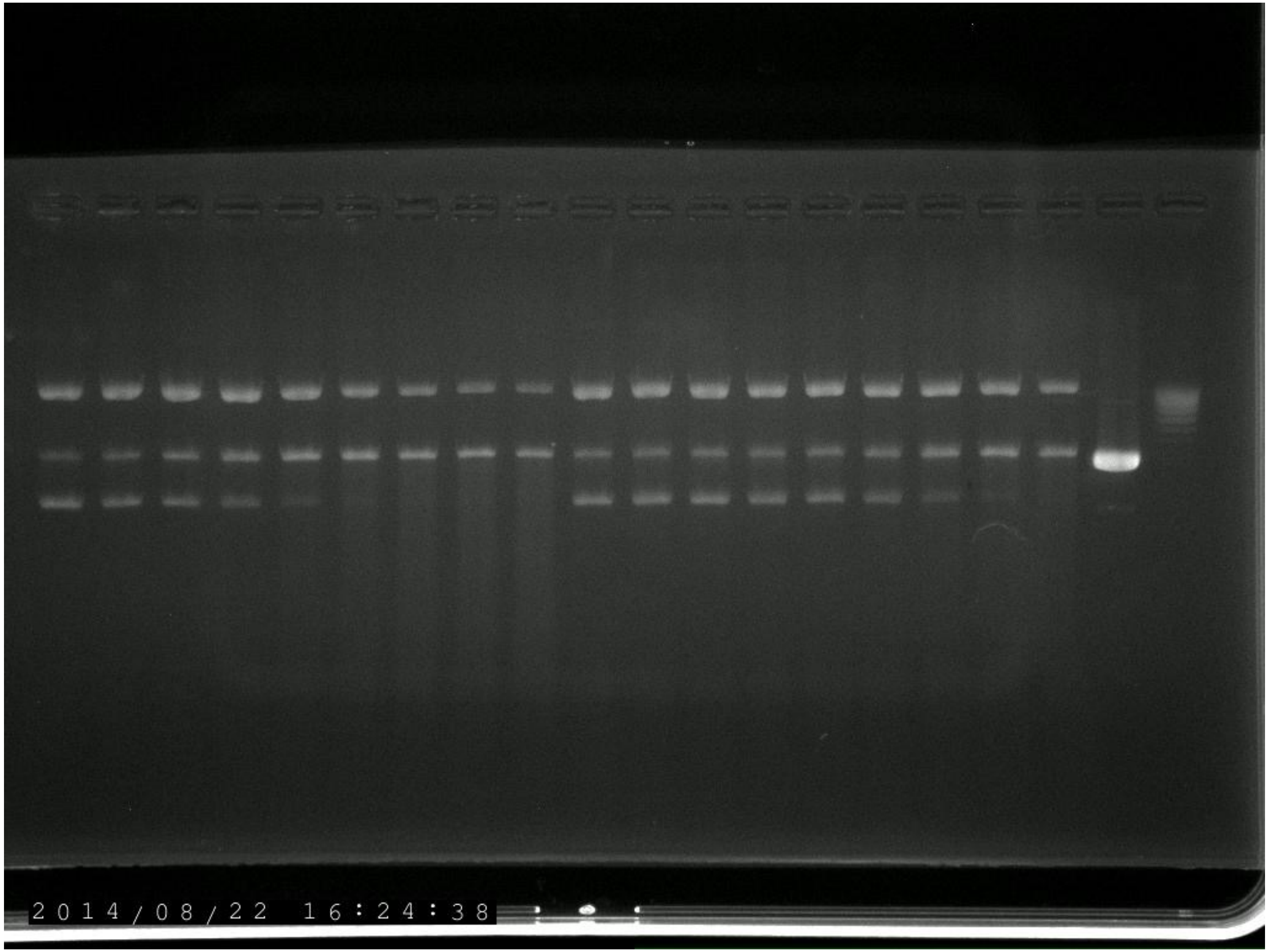

Fig 1b top →
(apigenin and
CID12261165)

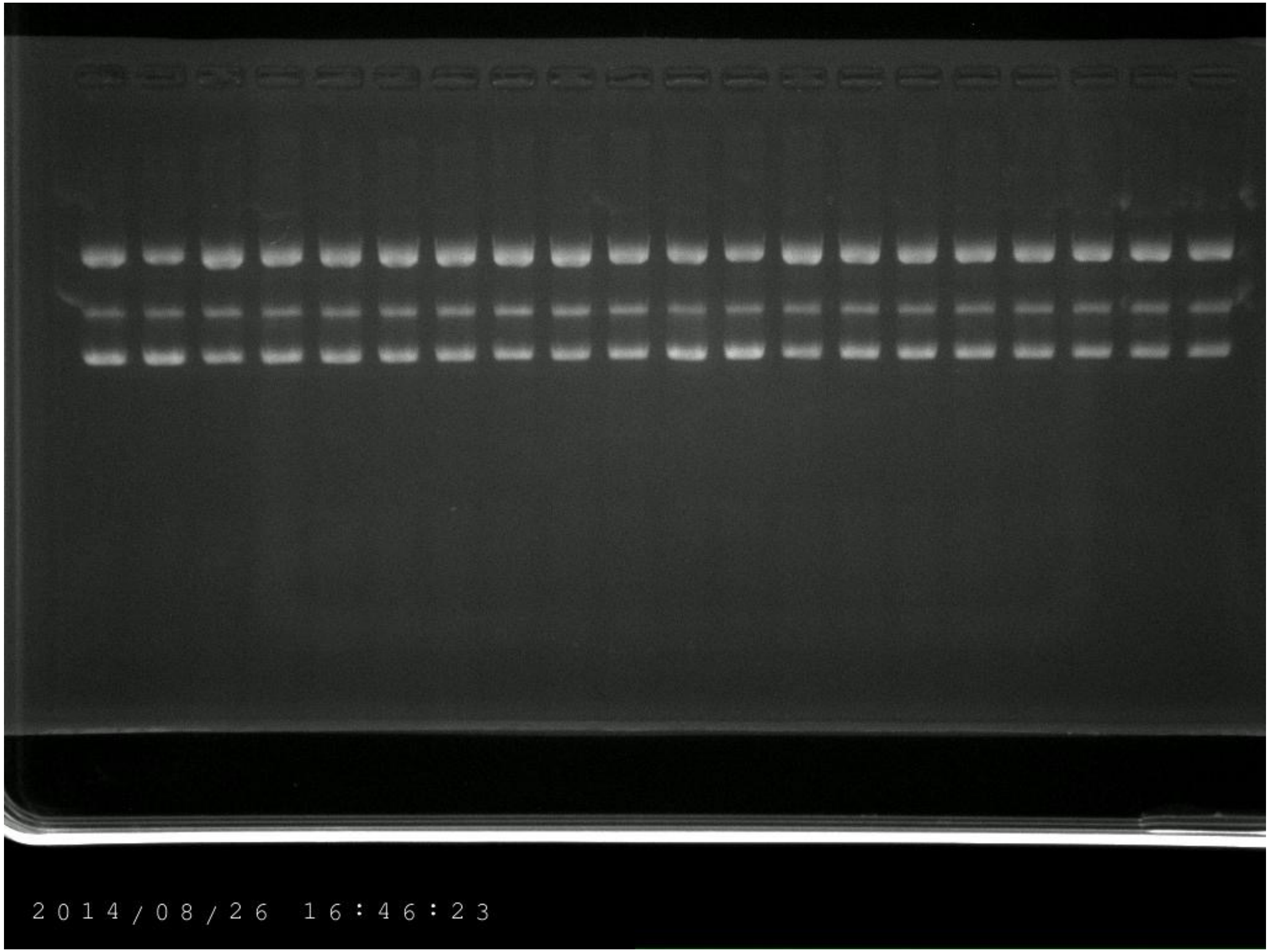

Fig 1c top →
(CID12261165)

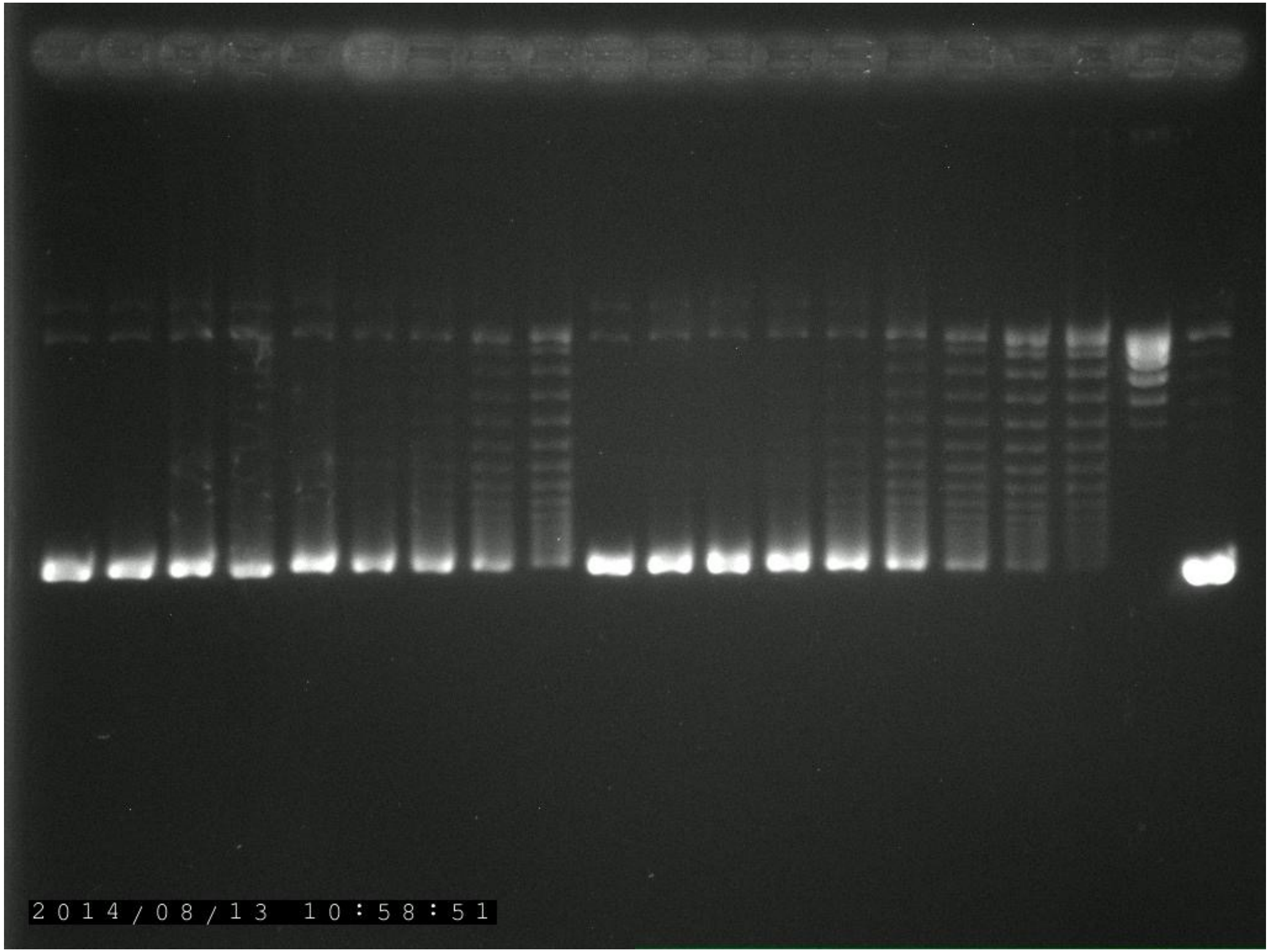

Fig 1c middle (apigenin) →

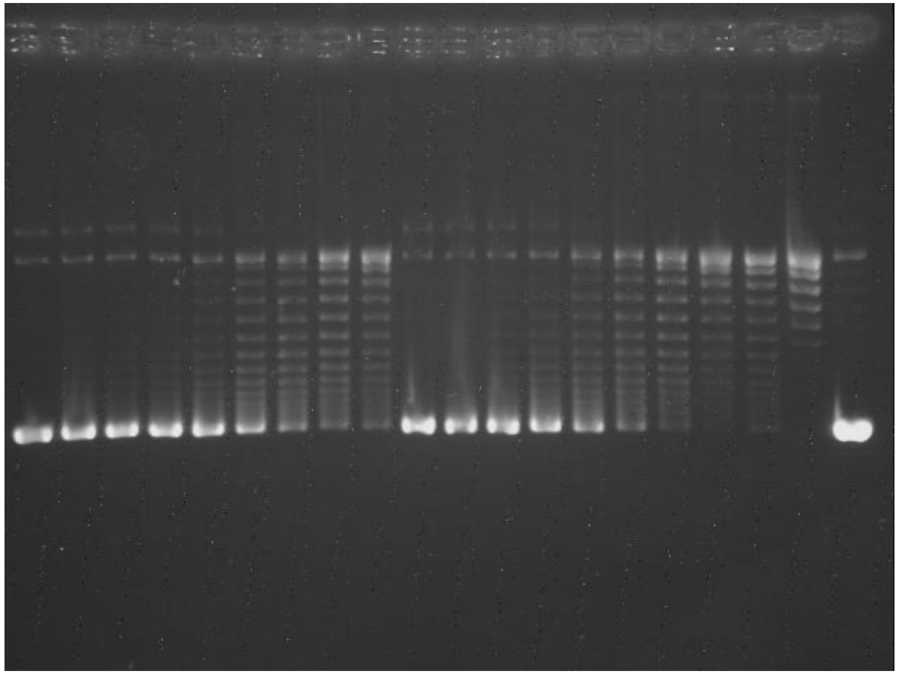

Fig 1c middle (levofloxacin) →

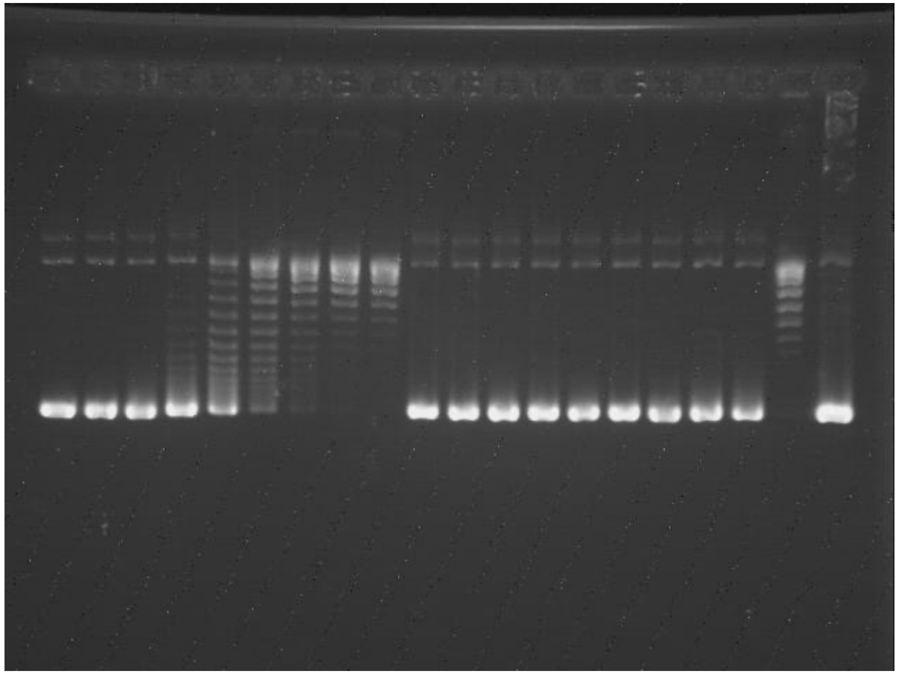

Supplement: Supplementary file 1 — Supplementary Information 1. [file 41598_2023_28859_MOESM1_ESM.pdf]
