# Supplementary material for: CID12261165, a flavonoid compound as antibacterial agents against quinolone-resistant Staphylococcus aureus
Source: Sci Rep. 2023 Jan 31;13:1725. doi: 10.1038/s41598-023-28859-8 (PMC9889749; doi:10.1038/s41598-023-28859-8)
Supplement: Supplementary file 2 — Supplementary Tables. [file 41598_2023_28859_MOESM2_ESM.pdf]

Table S1 Doubling time of MR5867 and quinolone stepwise mutants

Strain	Try1	Try2	Try3	average	SD	Standard Error
MS5867-P	28.30	27.02	27.21	27.51	0.566	0.326689373
MS5867-1	35.02	34.45	33.49	34.32	0.628	0.362614699
MS5867-2	32.34	27.31	35.03	31.56	3.197	1.845715699
MS5867-3	29.18	27.00	28.29	28.16	0.894	0.516235615
MS5867-4	27.50	25.05	27.51	26.68	1.157	0.668113901

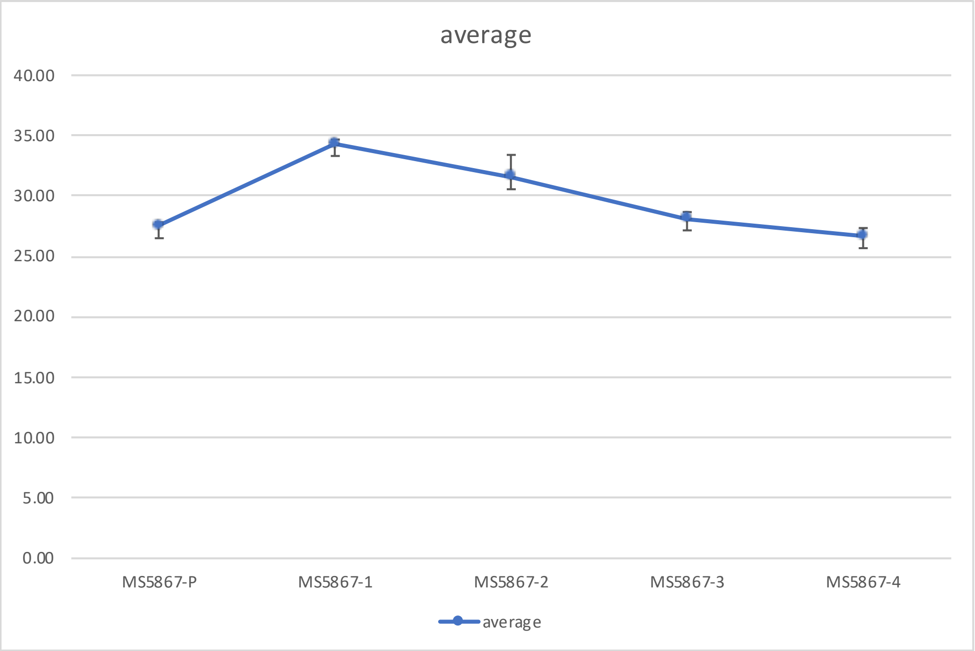

Table S2 Mutant selection using CID12261165 against quinolone resistant *S. aureus* strain Mu50

	Number of colonies*	Inoculum size (CFU/plate)	Appearance rate	*parC*	*gyrA*	MIC	
						M246	LVFX
Mu50	-		-	S80F	S84L	4	8
1	14	1.46×10^{8}	9.59×10^{-8}	S80F	S84L, L188S	64	8
2	9	1.08×10^{8}	8.33×10^{-8}	S80F	S84L, S98I	128	8
3	15	1.28×10^{8}	1.17×10^{-7}	S80F	S84L, P36S	64	16
4	3	1.07×10^{8}	2.80×10^{-8}	S80F	S84L, V29I	64	8
5	15	1.01×10^{8}	1.49×10^{-7}	S80F	S84L, E20L	128	16
6	11	1.90×10^{7}	5.79×10^{-7}	S80F	S84L, Q267P	64	8
7	10	1.18×10^{8}	8.48×10^{-8}	S80F	S84L, G41A	64	16
8	14	6.90×10^{7}	2.03×10^{-7}	S80F	S84L, S98I	128	8
9	6	1.10×10^{8}	5.46×10^{-8}	S80F	S84L, S98I	64	16
10	9	1.78×10^{8}	5.06×10^{-8}	S80F	S84L, S98I	128	16

*Number of colonies appeared on the mutant selection plate with CID12261165 concentration of 32mg/L
