# Supplementary material for: CID12261165, a flavonoid compound as antibacterial agents against quinolone-resistant Staphylococcus aureus
Source: Sci Rep. 2023 Jan 31;13:1725. doi: 10.1038/s41598-023-28859-8 (PMC9889749; doi:10.1038/s41598-023-28859-8)
Supplement: Supplementary file 3 — Supplementary Figure 1. [file 41598_2023_28859_MOESM3_ESM.pdf]

Supplemental Figure 1. Chemical structure of apigenin and five tested flavonoids

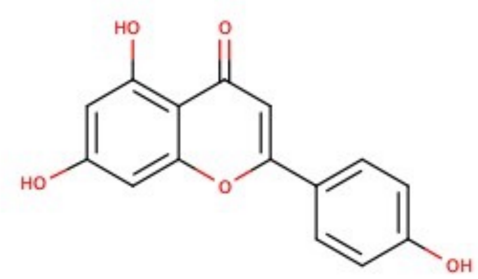


Apigenin

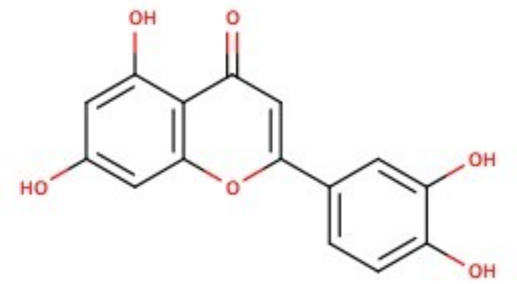


Luteolin

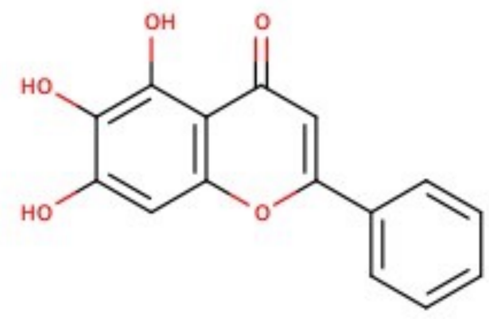


Baicalein

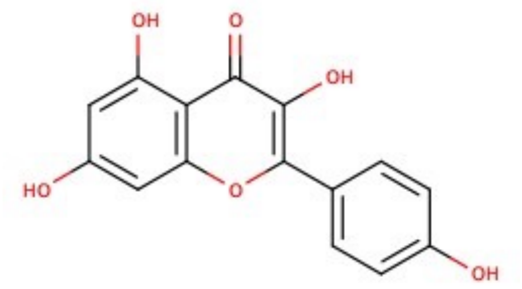


Kaempferol

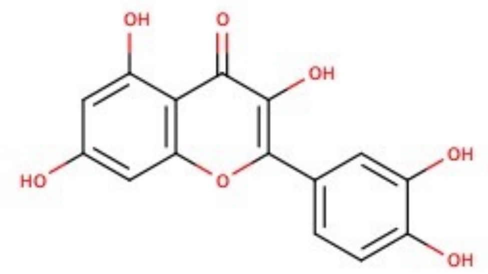


Quercetin

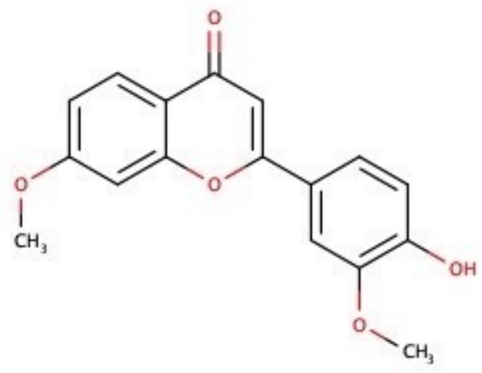


CID12261165
