# Supplementary material for: CID12261165, a flavonoid compound as antibacterial agents against quinolone-resistant Staphylococcus aureus
Source: Sci Rep. 2023 Jan 31;13:1725. doi: 10.1038/s41598-023-28859-8 (PMC9889749; doi:10.1038/s41598-023-28859-8)
Supplement: Supplementary file 4 — Supplementary Information 2. [file 41598_2023_28859_MOESM4_ESM.pdf]

In silico pharmacological safety assessment report

Software name and version:

ACD/Percepta 14.3.0 (Build 3063) (ACD/Lab, Canada)

Samples:

CID12261165, quercetin, luteolin, kaempferol, baicalein, apigenin, levofloxacin

Table of Contents

1. Cytochrome P450 inhibitors module reports ·················· P2
2. Protein binding module reports ······························ P9
3. Ames test module reports ···································· P16
4. hERG test reports ··· P23

ACD/Labs

P450 Inhibitors Module Report

Date: December 17, 2022 9:35 AM

Software name and version: ACD/Percepta 14.3.0 (Build 3063)

Compound name:

Structure:

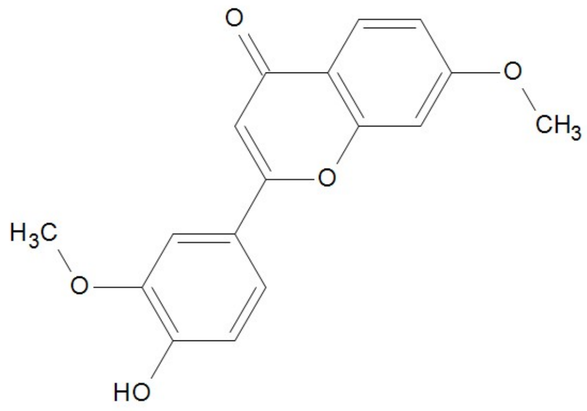

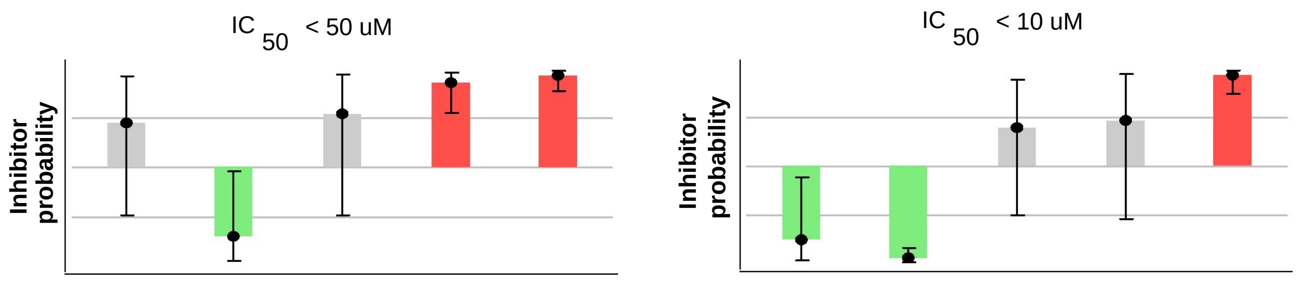

Cytochrome P450 isoforms

		CYP3A4	CYP2D6	CYP2C9	CYP2C19	CYP1A2
**IC_{50} < 50 uM**	Probability	0.71	0.15	0.77	0.92	0.95
	Reliability	0.42	0.55	0.32	0.72	0.78

		CYP3A4	CYP2D6	CYP2C9	CYP2C19	CYP1A2
**IC_{50} < 10 uM**	Probability	0.12	0.04	0.69	0.73	0.96
	Reliability	0.52	0.81	0.45	0.34	0.72

IC_{50} estimated at substrate concentration close to Km

ACD/Labs

P450 Inhibitors Module Report

Date: December 17, 2022 9:35 AM

Software name and version: ACD/Percepta 14.3.0 (Build 3063)

Compound name: Quercetin

Structure:

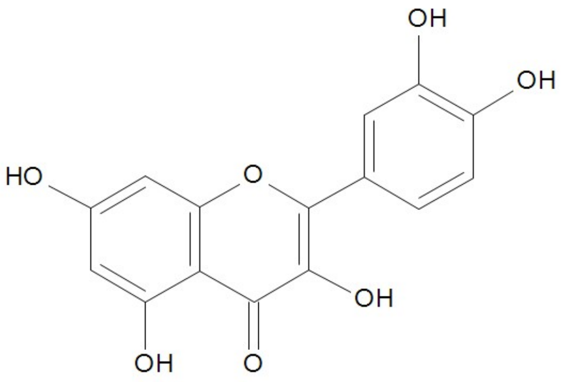

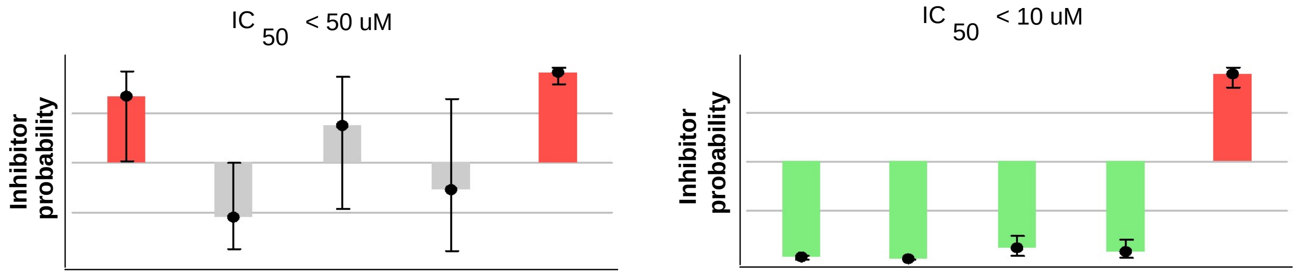

Cytochrome P450 isoforms

IC_{50} < 50 uM		CYP3A4	CYP2D6	CYP2C9	CYP2C19	CYP1A2
	Probability	0.83	0.23	0.68	0.37	0.95
	Reliability	0.59	0.71	0.50	0.38	0.89

IC_{50} < 10 uM		CYP3A4	CYP2D6	CYP2C9	CYP2C19	CYP1A2
	Probability	0.02	0.00	0.07	0.05	0.94
	Reliability	0.97	0.98	0.89	0.83	0.88

IC_{50} estimated at substrate concentration close to Km

ACD/Labs

P450 Inhibitors Module Report

Date: December 17, 2022 9:35 AM

Software name and version: ACD/Percepta 14.3.0 (Build 3063)

Compound name: Luteolin

Structure:

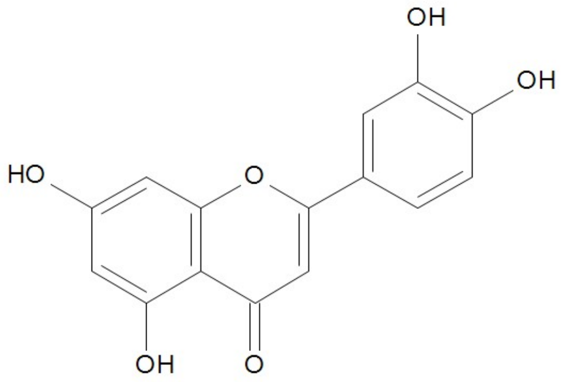

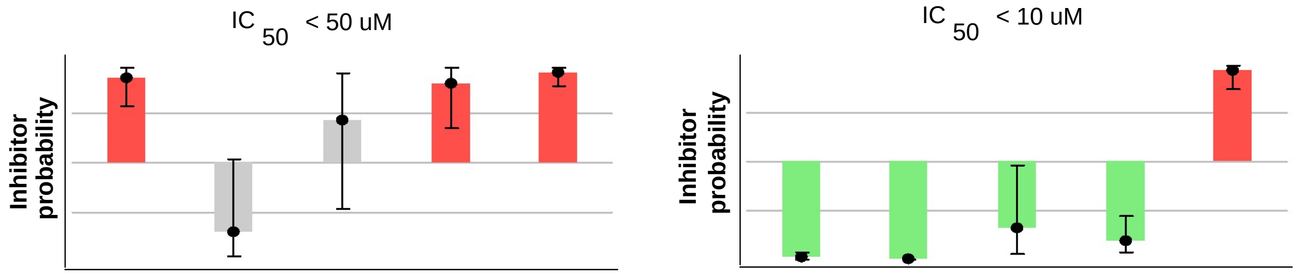

Cytochrome P450 isoforms

IC_{50} < 50 uM		CYP3A4	CYP2D6	CYP2C9	CYP2C19	CYP1A2
	Probability	0.92	0.15	0.71	0.89	0.95
	Reliability	0.73	0.49	0.44	0.65	0.86

IC_{50} < 10 uM		CYP3A4	CYP2D6	CYP2C9	CYP2C19	CYP1A2
	Probability	0.02	0.00	0.17	0.10	0.96
	Reliability	0.88	0.96	0.58	0.81	0.73

IC_{50} estimated at substrate concentration close to Km

Date: December 17, 2022 9:34 AM

Software name and version: ACD/Percepta 14.3.0 (Build 3063)

Compound name: Kaempferol

Structure:

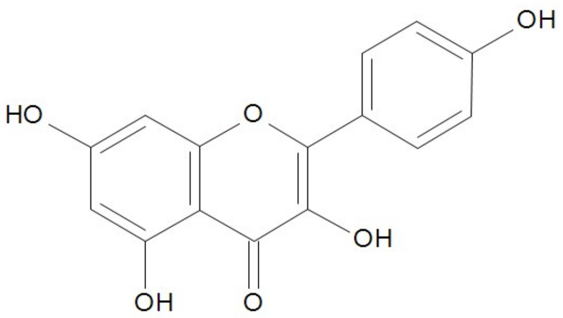

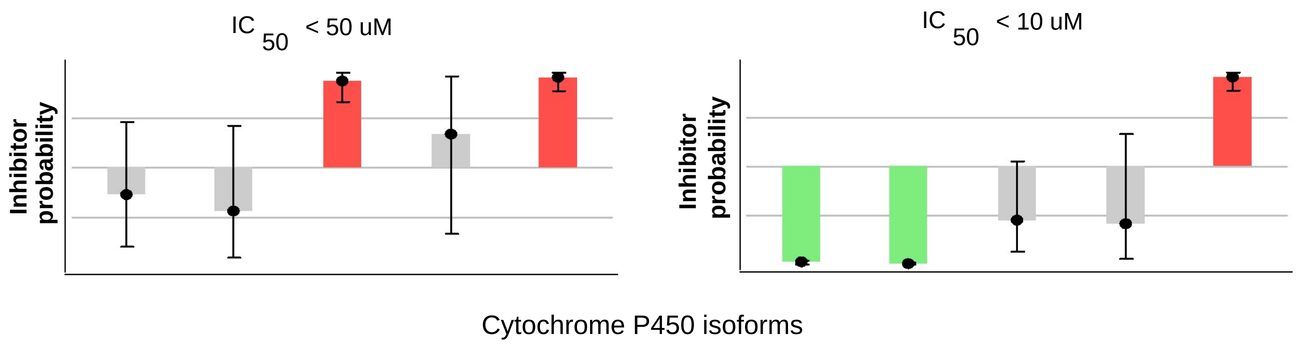

		CYP3A4	CYP2D6	CYP2C9	CYP2C19	CYP1A2
**IC_{50} < 50 uM**	Probability	0.36	0.28	0.93	0.66	0.94
	Reliability	0.59	0.47	0.81	0.30	0.88

		CYP3A4	CYP2D6	CYP2C9	CYP2C19	CYP1A2
**IC_{50} < 10 uM**	Probability	0.02	0.01	0.23	0.21	0.94
	Reliability	0.96	0.97	0.66	0.39	0.89

IC_{50} estimated at substrate concentration close to Km

ACD/Labs

P450 Inhibitors Module Report

Date: December 17, 2022 9:35 AM

Software name and version: ACD/Percepta 14.3.0 (Build 3063)

Compound name: Baicalein

Structure:

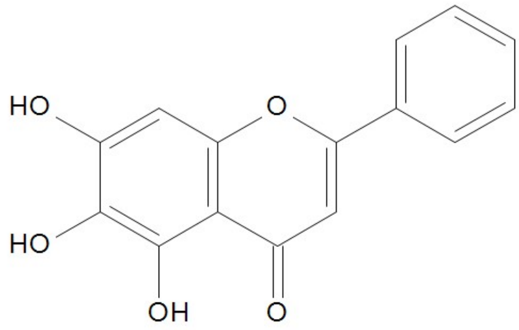

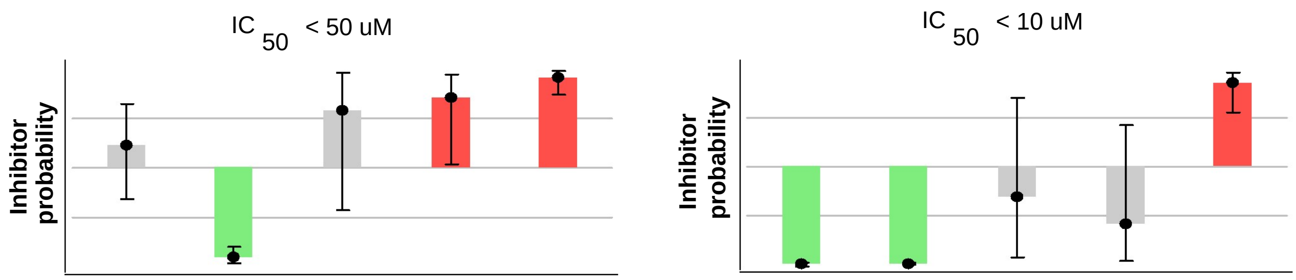

Cytochrome P450 isoforms

**IC_{50} < 50 uM**		CYP3A4	CYP2D6	CYP2C9	CYP2C19	CYP1A2
	Probability	0.61	0.05	0.78	0.84	0.94
	Reliability	0.77	0.87	0.34	0.55	0.80

**IC_{50} < 10 uM**		CYP3A4	CYP2D6	CYP2C9	CYP2C19	CYP1A2
	Probability	0.01	0.01	0.34	0.21	0.92
	Reliability	0.94	0.94	0.26	0.31	0.70

IC_{50} estimated at substrate concentration close to Km

Date: December 17, 2022 9:34 AM

Software name and version: ACD/Percepta 14.3.0 (Build 3063)

Compound name: Apigenin

Structure:

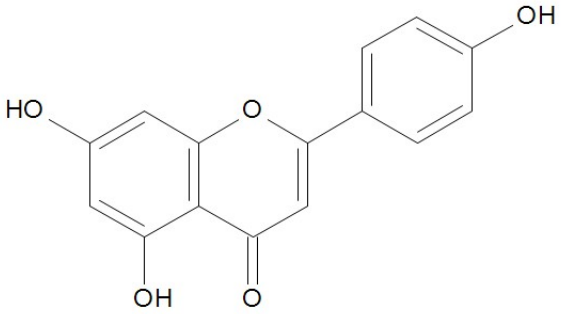

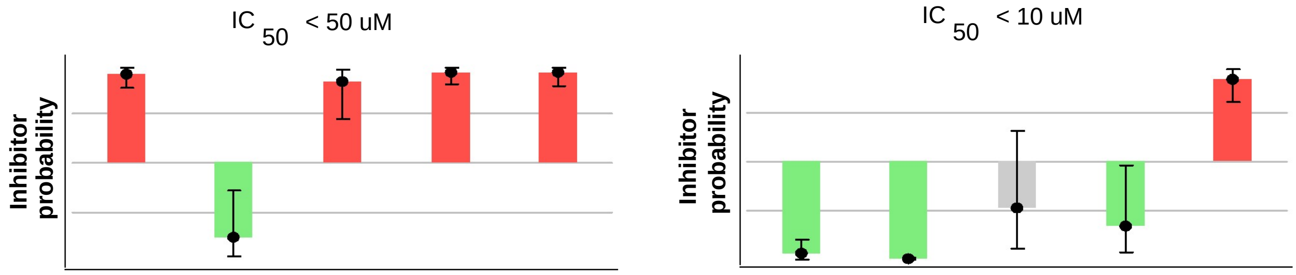

Cytochrome P450 isoforms

		CYP3A4	CYP2D6	CYP2C9	CYP2C19	CYP1A2
**IC_{50} < 50 uM**	Probability	0.93	0.13	0.90	0.94	0.94
	Reliability	0.89	0.67	0.69	0.92	0.88

		CYP3A4	CYP2D6	CYP2C9	CYP2C19	CYP1A2
**IC_{50} < 10 uM**	Probability	0.03	0.01	0.26	0.17	0.91
	Reliability	0.70	0.97	0.54	0.61	0.82

IC_{50} estimated at substrate concentration close to Km

Date: December 17, 2022 9:34 AM

Software name and version: ACD/Percepta 14.3.0 (Build 3063)

Compound name: Levofloxacin

Structure:

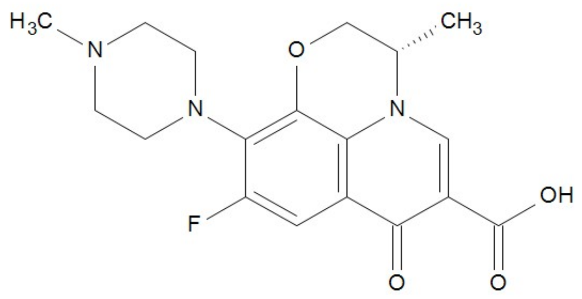

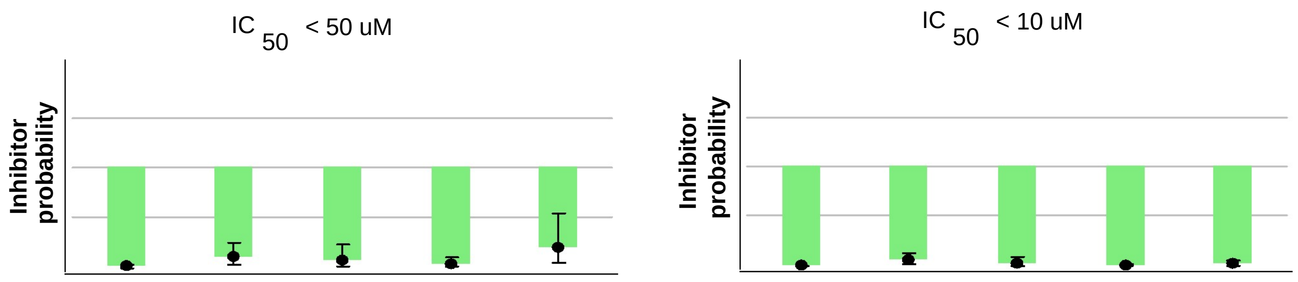

Cytochrome P450 isoforms

**IC_{50} < 50 uM**		CYP3A4	CYP2D6	CYP2C9	CYP2C19	CYP1A2
	Probability	0.01	0.06	0.03	0.02	0.10
	Reliability	0.93	0.83	0.67	0.79	0.71

**IC_{50} < 10 uM**		CYP3A4	CYP2D6	CYP2C9	CYP2C19	CYP1A2
	Probability	0.00	0.02	0.01	0.00	0.01
	Reliability	0.90	0.82	0.68	0.80	0.85

IC_{50} estimated at substrate concentration close to Km

Date: December 17, 2022 10:27 AM

Software name and version: ACD/Percepta 14.3.0 (Build 3063)

Compound name:

Structure:

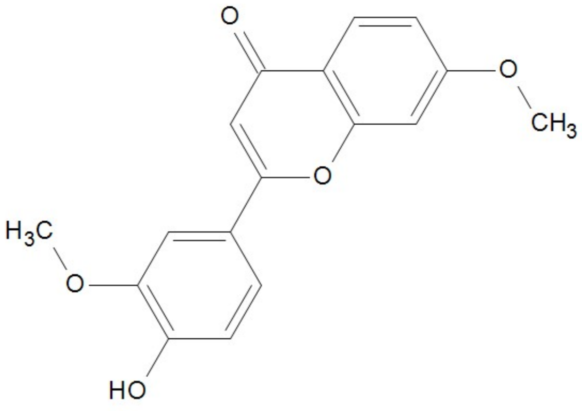

%PPB: 91.03%

Reliability: Moderate (RI = 0.52)

Library used in calculation:

%PPB v. 1.2 (Read-only)

$LogK_a^{HSA}$: 3.99

Reliability: Borderline (RI = 0.34)

Library used in calculation:

LogKa(HSA) v. 1.2 (Read-only)

Zwitterionic compound. These drugs are likely to bind to the majority of plasma proteins.

ACD/Labs

Date: December 17, 2022 10:27 AM

Software name and version: ACD/Percepta 14.3.0 (Build 3063)

Compound name: Quercetin

Structure:

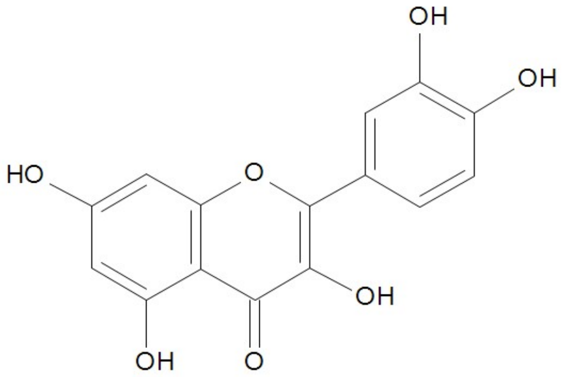

%PPB: 93.38%

Reliability: High (RI = 0.77)

Library used in calculation:
%PPB v. 1.2 (Read-only)

$LogK_a^{HSA}$: 4.06

Reliability: Not Reliable (RI = 0.28)

Library used in calculation:
LogKa(HSA) v. 1.2 (Read-only)

Acidic compound. In plasma these drugs predominantly bind to human serum albumin.

Date: December 17, 2022 10:26 AM

Software name and version: ACD/Percepta 14.3.0 (Build 3063)

Compound name: Luteolin

Structure:

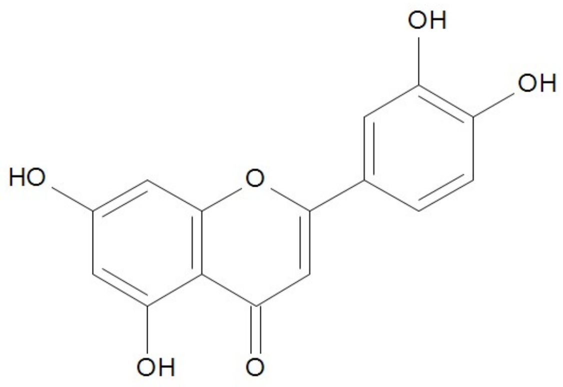

%PPB: 93.51%

Reliability: High (RI = 0.77)

Library used in calculation:

%PPB v. 1.2 (Read-only)

$LogK_a^{HSA}$: 4.02

Reliability: Not Reliable (RI = 0.29)

Library used in calculation:

LogKa(HSA) v. 1.2 (Read-only)

Zwitterionic compound. These drugs are likely to bind to the majority of plasma proteins.

Date: December 17, 2022 10:27 AM

Software name and version: ACD/Percepta 14.3.0 (Build 3063)

Compound name: Kaempferol

Structure:

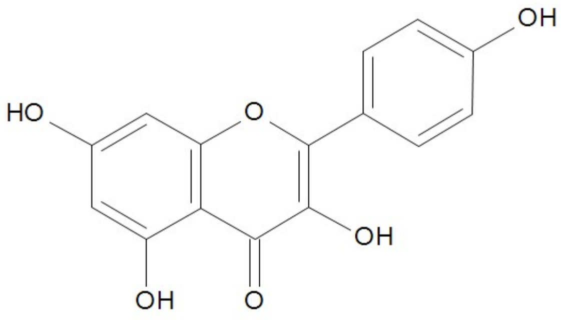

%PPB: 93.54%

Reliability: Moderate (RI = 0.63)

Library used in calculation:

%PPB v. 1.2 (Read-only)

LogK$_a^{HSA}$: 4.49

Reliability: Borderline (RI = 0.33)

Library used in calculation:

LogKa(HSA) v. 1.2 (Read-only)

Acidic compound. In plasma these drugs predominantly bind to human serum albumin.

Date: December 17, 2022 10:26 AM

Software name and version: ACD/Percepta 14.3.0 (Build 3063)

Compound name: Baicalein

Structure:

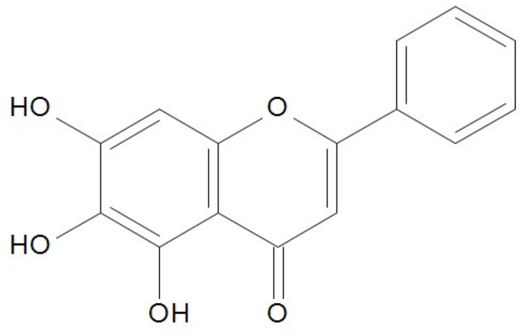

%PPB: 90.94%

Reliability: Moderate (RI = 0.58)

Library used in calculation:
%PPB v. 1.2 (Read-only)

$LogK_a^{HSA}$: 3.96

Reliability: Not Reliable (RI = 0.18)

Library used in calculation:
LogKa(HSA) v. 1.2 (Read-only)

Zwitterionic compound. These drugs are likely to bind to the majority of plasma proteins.

Date: December 17, 2022 10:25 AM

Software name and version: ACD/Percepta 14.3.0 (Build 3063)

Compound name: Apigenin

Structure:

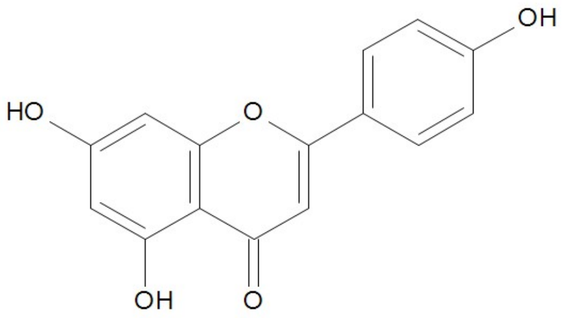

%PPB: 94.02%

Reliability: Moderate (RI = 0.57)

Library used in calculation:

%PPB v. 1.2 (Read-only)

$LogK_a^{HSA}$: 4.53

Reliability: Borderline (RI = 0.33)

Library used in calculation:

LogKa(HSA) v. 1.2 (Read-only)

Zwitterionic compound. These drugs are likely to bind to the majority of plasma proteins.

Date: December 17, 2022 10:28 AM

Software name and version: ACD/Percepta 14.3.0 (Build 3063)

Compound name: Levofloxacin

Structure:

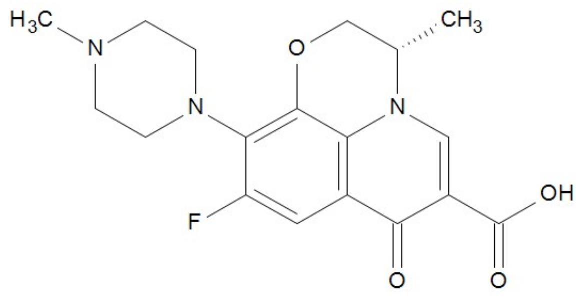

%PPB: 32.89%

Reliability: High (RI = 0.88)

Library used in calculation:
%PPB v. 1.2 (Read-only)

$LogK_a^{HSA}$: 4.17

Reliability: High (RI = 0.79)

Library used in calculation:
LogKa(HSA) v. 1.2 (Read-only)

Zwitterionic compound. These drugs are likely to bind to the majority of plasma proteins.

Date: December 17, 2022 9:21 AM

Software name and version: ACD/Percepta 14.3.0 (Build 3063)

Compound name:

Structure:

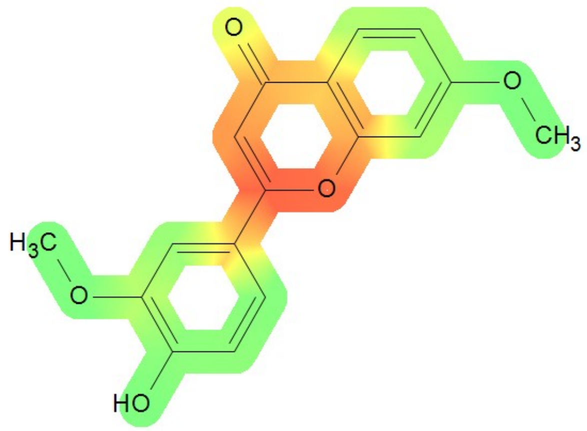

Probability of positive Ames test: 0.82

Reliability: Borderline (RI = 0.39)

Library used in calculation:

AMES Test v. 1.2 (Read-only)

Date: December 17, 2022 9:22 AM

Software name and version: ACD/Percepta 14.3.0 (Build 3063)

Compound name: Quercetin

Structure:

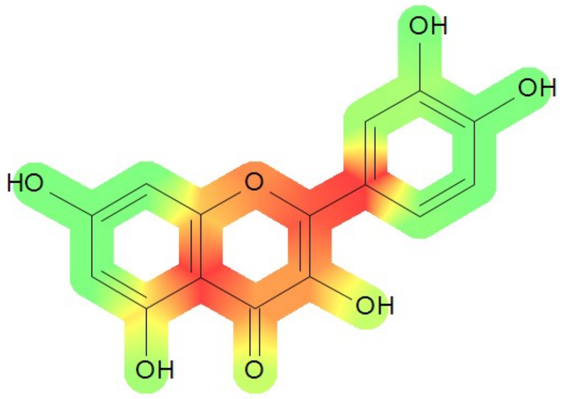

Probability of positive Ames test: 0.85

Reliability: Borderline (RI = 0.48)

Library used in calculation:

AMES Test v. 1.2 (Read-only)

ACD/Labs

Ames Test Module Report

Date: December 17, 2022 9:22 AM

Software name and version: ACD/Percepta 14.3.0 (Build 3063)

Compound name: Luteolin

Structure:

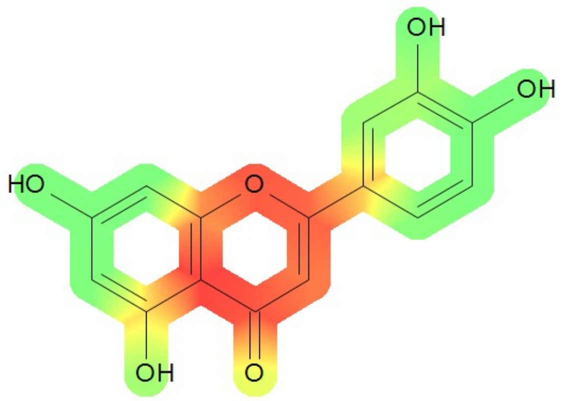

Probability of positive Ames test: 0.48

Reliability: Borderline (RI = 0.49)

Library used in calculation:

AMES Test v. 1.2 (Read-only)

Date: December 17, 2022 9:23 AM

Software name and version: ACD/Percepta 14.3.0 (Build 3063)

Compound name: Kaempferol

Structure:

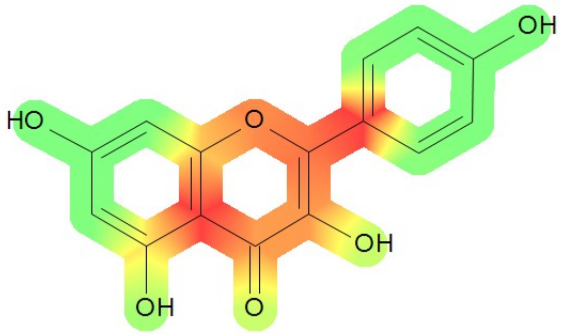

Probability of positive Ames test: 0.92

Reliability: High (RI = 0.87)

Library used in calculation:

AMES Test v. 1.2 (Read-only)

Date: December 17, 2022 9:22 AM

Software name and version: ACD/Percepta 14.3.0 (Build 3063)

Compound name: Baicalein

Structure:

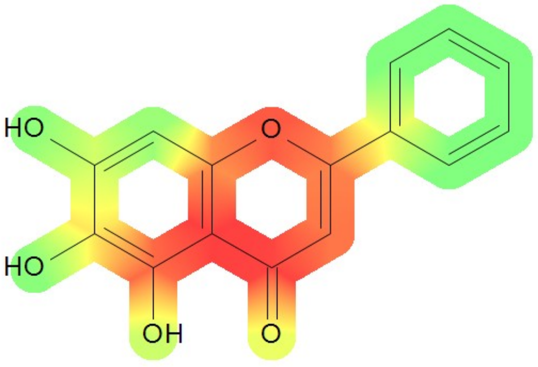

Probability of positive Ames test: 0.94

Reliability: High (RI = 0.9)

Library used in calculation:

AMES Test v. 1.2 (Read-only)

Date: December 17, 2022 9:23 AM

Software name and version: ACD/Percepta 14.3.0 (Build 3063)

Compound name: Apigenin

Structure:

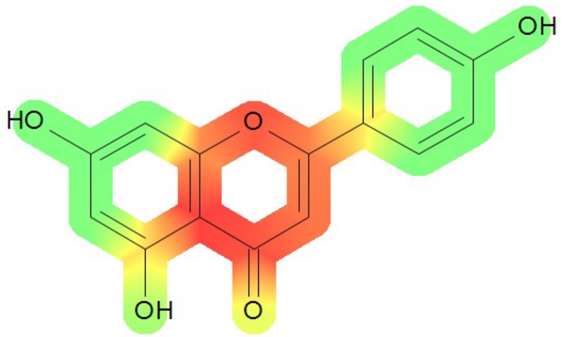

Probability of positive Ames test: 0.47

Reliability: Moderate (RI = 0.59)

Library used in calculation:

AMES Test v. 1.2 (Read-only)

Date: December 17, 2022 9:23 AM

Software name and version: ACD/Percepta 14.3.0 (Build 3063)

Compound name: Levofloxacin

Structure:

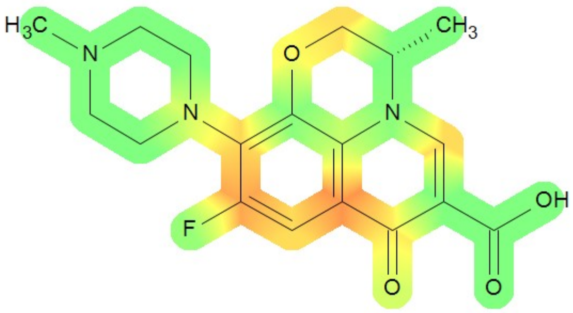

Probability of positive Ames test: 0.46

Reliability: Moderate (RI = 0.68)

Library used in calculation:

AMES Test v. 1.2 (Read-only)

Date: December 17, 2022 9:25 AM

Software name and version: ACD/Percepta 14.3.0 (Build 3063)
Compound name:
Structure:

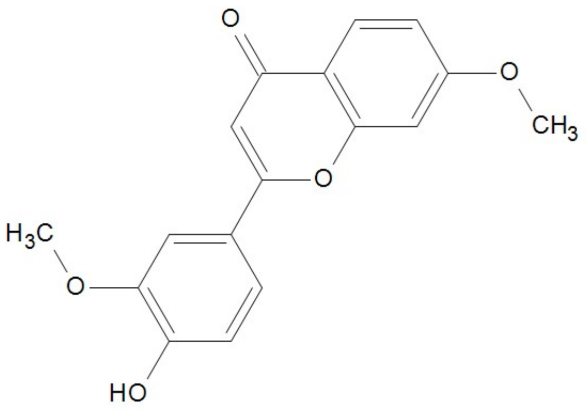

hERG inhibitor (Ki < 10 uM, patch-clamp) probability: 0.04

Reliability: Borderline (RI = 0.45)

Library used in calculation:
hERG-I (Ki less than 10 uM) v. 1.3 (Read-only)

Date: December 17, 2022 9:25 AM

Software name and version: ACD/Percepta 14.3.0 (Build 3063)

Compound name: Quercetin

Structure:

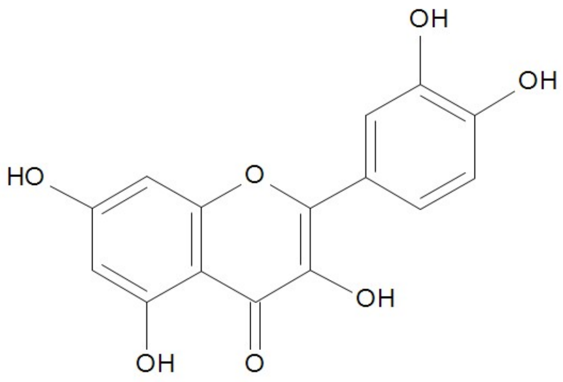

hERG inhibitor (Ki < 10 uM, patch-clamp) probability: 0.00

Reliability: Borderline (RI = 0.49)

Library used in calculation:

hERG-I (Ki less than 10 uM) v. 1.3 (Read-only)

Date: December 17, 2022 9:25 AM

Software name and version: ACD/Percepta 14.3.0 (Build 3063)

Compound name: Luteolin

Structure:

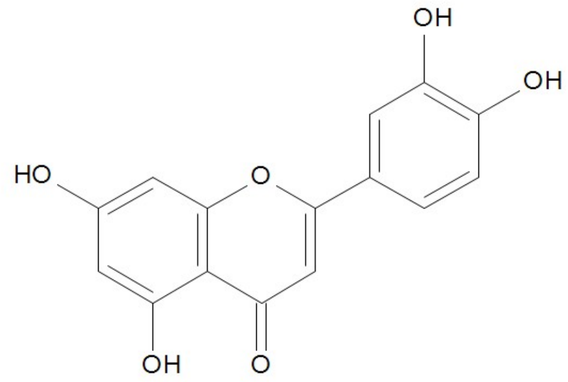

hERG inhibitor (Ki < 10 uM, patch-clamp) probability: 0.00

Reliability: Borderline (RI = 0.41)

Library used in calculation:

hERG-I (Ki less than 10 uM) v. 1.3 (Read-only)

Date: December 17, 2022 9:25 AM

Software name and version: ACD/Percepta 14.3.0 (Build 3063)
Compound name: Kaempferol
Structure:

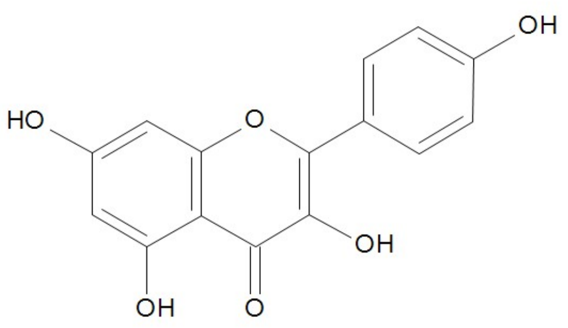

hERG inhibitor (Ki < 10 uM, patch-clamp) probability: 0.00

Reliability: Moderate (RI = 0.57)

Library used in calculation:
hERG-I (Ki less than 10 uM) v. 1.3 (Read-only)

Date: December 17, 2022 9:25 AM

Software name and version: ACD/Percepta 14.3.0 (Build 3063)
Compound name: Baicalein
Structure:

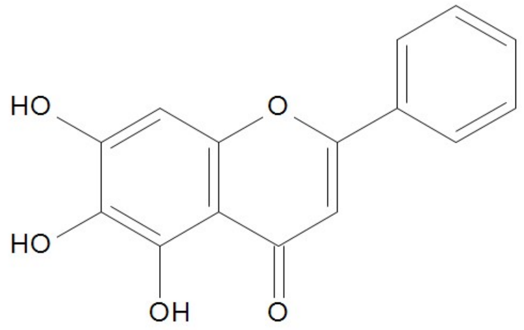

hERG inhibitor (Ki < 10 uM, patch-clamp) probability: 0.00

Reliability: High (RI = 0.83)

Library used in calculation:
hERG-I (Ki less than 10 uM) v. 1.3 (Read-only)

ACD/Labs

hERG Inhibition Module Report

Date: December 17, 2022 9:24 AM

Software name and version: ACD/Percepta 14.3.0 (Build 3063)
Compound name: Apigenin
Structure:

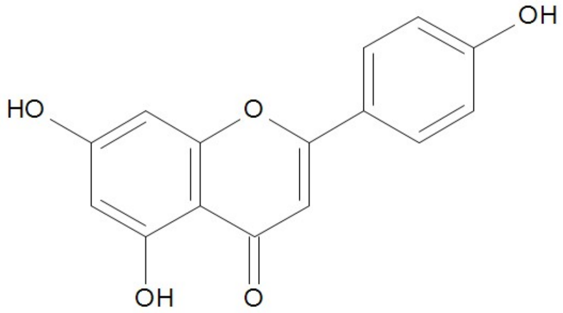

hERG inhibitor (Ki < 10 uM, patch-clamp) probability: 0.00

Reliability: High (RI = 0.83)

Library used in calculation:
hERG-I (Ki less than 10 uM) v. 1.3 (Read-only)

ACD/Labs

hERG Inhibition Module Report

Date: December 17, 2022 9:25 AM

Software name and version: ACD/Percepta 14.3.0 (Build 3063)

Compound name: Levofloxacin

Structure:

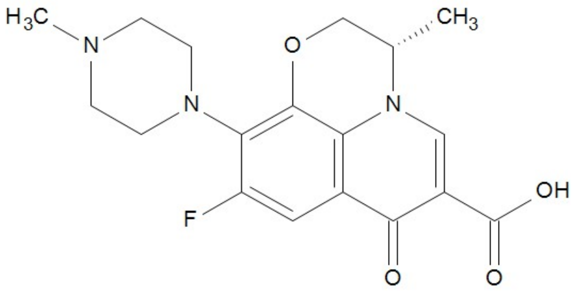

hERG inhibitor (Ki < 10 uM, patch-clamp) probability: 0.06

Reliability: High (RI = 0.86)

Library used in calculation:

hERG-I (Ki less than 10 uM) v. 1.3 (Read-only)
